# Ac-YVAD-cmk ameliorated sevoflurane-induced cognitive dysfunction and revised mitophagy impairment

**DOI:** 10.1371/journal.pone.0280914

**Published:** 2023-01-25

**Authors:** Du Zheng, Hongwei Wang, Youfa Zhou, Yeru Chen, Gang Chen

**Affiliations:** Department of Anesthesiology, Sir Run Run Shaw Hospital, School of Medicine, Zhejiang University, Hangzhou, Zhejiang, China; University College London, UNITED KINGDOM

## Abstract

It is common for elderly patients to develop postoperative cognitive dysfunction (POCD), but the pathophysiological mechanisms have not yet been fully explored. NLRP3 inflammasome activation and mitophagy impairment was involved in neurodegenerative disease. This study investigated the interaction of NLRP3 inflammasome and mitophagy in sevoflurane-induced cognitive dysfunction. We found that sevoflurane induced cleaved caspase-1 level, IL-1β and IL-18 maturation, and activated NLRP3 inflammasome in aged mice and the primary hippocampus neuron. The cleaved caspase-1 was demonstrated in microglia of hippocampus. Ac-YVAD-cmk, a selected caspase-1 inhibitor, reduced the expression of cleaved caspase-1, IL-1β, IL-18 and NLRP3 inflammasome activation induced by sevoflurane. Ac-YVAD-cmk ameliorated learning ability impairment in aged mice induced by sevoflurane using Morris water maze. Moreover, Ac-YVAD-cmk reversed the mitophagy flux dysfunction induced by sevoflurane in aged mice by western blotting, immunostaining and mt-Keima reporters. For the first time, we found caspase-1 inhibitor mitigated mitochondria dysfunction and revised mitophagy impairment induced by sevoflurane.

## Introduction

The first research reported the relationship between surgery under general anesthesia and postoperative cognitive dysfunction (POCD) in 1955 [1]. There is a significant reduction in cognitive function in POCD patients, which can last for up to six months or even longer [2]. As the most commonly used anesthetic, sevoflurane anesthesia was reported to be associated with cognitive impairment [3]. Cognitive dysfunction induced by sevoflurane may also be caused by neuroinflammation [4], autophagy [5], oxidative stress [6], blood-brain barrier dysfunction [7], and apoptosis [8]. The underlying mechanisms of sevoflurane-induced cognitive impairment remained unknown.

Increasing evidences revealed a causal relationship between sevoflurane-induced cognitive impairment and NLRP3 inflammasomes in hippocampus [9,10]. In response to cellular stress, NLRP3 recruits ASC and pro-caspase-1, which results in cleaved caspase-1 activation and processing of the maturation of IL-1β and IL-18 [11]. Patients with Alzheimer’s disease exhibit highly enhanced active caspase-1 expression in their brains, suggesting that the inflammasome plays a role in this neurodegenerative disease [9]. In addition, as an important way for elimination of damaged mitochondrial, mitophagy reduces cellular stress playing a crucial role in neurodegenerative disease and aging [12,13]. Our previous work indicated that mitophagy impairment contributes to sevoflurane-induced cognitive dysfunction in aged rats [14]. However, the interaction between NLRP3 inflammasomes and mitophagy in the sevoflurane-induced cognitive impairment had not been fully clarified.

Numerous danger signals activate the NLRP3 inflammasome. A model suggests that NLRP3 is activated by ROS produced in mitochondria. By producing ROS, mitochondria with reduced membrane potential activate NLRP3 inflammasomes [15]. However, induction of mitochondrial outer membrane permeabilization and mitochondrial permeability transition by danger-associated molecular patterns (DAMP), inflammasome activation might leads to block of mitophagy. In present study, we used the caspase-1 inhibitor, Ac-YVAD-cmk to explore the neuroprotection on hippocampus, and to explain the mechanisms underlying the relationship between NLRP3 inflammasomes and mitophagy.

## Materials and methods

### Animals

Zhejiang Science Laboratory Animal Welfare Ethics Review Committee approved this project. (No. ZJU20160074) We conducted all experiments based on its ethical guidelines and in compliance with the National Institutes of Health Guide for the Care and Use of Laboratory Animals and in accordance with ARRIVE guidelines. Eighteen-month-old mice were used in this study and obtained from Zhejiang Academy of Medical Sciences. A standard animal care facility provided food and water to these mice for a month, as well as a 12/12 hour light/dark cycle and a constant temperature of 22°C for the duration of the experiment. Our goal was to minimize pain and discomfort for the animals, and we used the smallest number of animals possible.

### Animal treatment

A sevoflurane vaporizer and an anesthetizing chamber with two connections was used to induce general anesthesia in aged mice The aged mice of sevoflurane group were exposed to 2% sevoflurane delivered by a humidified 30% O_2_ carrier gas for 5 h. According to Ac-YVAD-cmk administration, the aged mice were randomly assigned to 4 groups: control group (Ctrl), sevoflurane group (SEV), Ac-YVAD-cmk group (AC) and sevoflurane plus Ac-YVAD-cmk group (SEV+AC). The aged mice of SEV and SEV+AC groups were exposed with 2% sevoflurane for 5h. The aged mice of Ctrl and AC groups were exposed to the carrier gas without sevoflurane in the same period time. According to previous studies [16], the mice in the AC and SEV+AC groups were administrated with Ac-YVAD-cmk (12.5 μmol/kg, i.p.) 1 h before sevoflurane treatment. According to MCC950 administration, the aged mice were randomly assigned to 4 groups: control group (Ctrl), sevoflurane group (SEV), MCC950 group and sevoflurane plus MCC950 group (SEV+MCC950). The aged mice of SEV and SEV+MCC950 groups were exposed with 2% sevoflurane for 5h. The aged mice of Ctrl and MCC950 groups were exposed to the carrier gas without sevoflurane in the same period time. Aged mice were injected intraperitoneally (i.p.) with 50 mg/kg MCC950 or vehicle control (DMSO/PBS) 1 h before sevoflurane treatment according to previous study [17]. In order to ensure sufficient ventilation, a single sample of arterial blood was obtained at the end of sevoflurane anesthesia or sham exposure via cardiac puncture from 5 mice of each group. These mice were not used for any other part of the study. Using a blood gas analyzer, arterial carbon dioxide partial pressure (PaCO2), arterial oxygen pressure (PaO2), blood oxygen saturation (SaO2), and power of hydrogen (PH) were measured. (Kent Scientific Corp., Torrington, CT, USA). The levels of pH, PaCO2, PaO2, Glucose, and SaO2 are not significantly different among the groups. All the mice were sacrificed after behavior tests. The mice were anesthetized with ketamine (60mg/kg) and xylazine (5mg/kg) intraperitoneally, then perfused with 0.01 mol/L phosphate buffered saline (PBS, pH 7.2–7.4) followed by 4% paraformaldehyde in 0.1 mol/L PB through the ascending aorta in immunostaining assay. In the immunoblot assay, ATP assay and the lipid peroxidation malondialdehyde (MDA) assay, the mice were anesthetized with ketamine (60mg/kg) and xylazine (5mg/kg) intraperitoneally, then perfused with 0.01 mol/L phosphate buffered saline (PBS, pH 7.2–7.4), the hippocampus were removed into lysis buffer.

### Morris water maze test

The spatial memory abilities were measured by using the Morris Water Maze (MWM) 24 h after the sevoflurane exposure and Ac-YVAD-cmk administration. The protocol was completed in according to previous reports [18]. A circular black pool (diameter: 120 cm; depth: 21 cm) was filled with opaque water using white non-toxic ink to reach 1.0 cm above the platform surface (diameter, 10 cm), and the water temperature was kept at 22°C. In the training phase, all animals received four training trials per day for a total of four days. The mice were placed into the pool at a special starting position and allowed to discover the hidden platform for 60 s. Mice were guided to the platform if they could not locate the platform within 1 min. The latency time (the time to reach the hidden platform) was recorded for assessing the spatial learning. After each trial, the mice were wiped dry and a heat lamp was used to faster temperature recovering before returning to home cages. In the testing phase, the platform was removed, and each mouse was allowed to swim freely in the pool for 2 min. The platform crossing times and the quadrant time were recorded for measuring memory function.

### Novel object recognition test

The novel object recognition test (NOR) was performed as described previously [19]. Briefly, mice were allowed to explore the chamber freely for 15 min on the first day. Then the mice were exposed to two identical objects (A) for 5 min during the training phase. After 24h, the mice were exposed to the familiar object (A) and a novel object (B) for 5 min during the test phase. The total distance traveled for the first 15 min on the first day was recorded and the recognition index was calculated: A recognition index was calculated for each animal and reported as the ratio TB/(TA + TB), where TA = time spent exploring the familiar object A and TB = time spent exploring the novel object B. Exploration was defined as sniffing or touching the object with the nose or forepaws.

### Cell culture and treatment

The primary hippocampal neuronal culture was performed as described [20]. Briefly, the dissected hippocampus from E17 fetal mice was used. The primary hippocampal neurons were cultured for 20 days before treatment and harvested for subsequent detection.

H4 human neuroglioma cells, which were harvested from the China Center for Type Culture Collection, were cultured in Dulbecco’s Modified Eagle’s Medium (DMEM) containing 10% heat-inactivated fetal bovine serum and 10% F12 (all from Gibco, Grand Island, NY, USA) at 37°C with 5% CO_2_ in a humidified incubator. Some previous reports indicated that 4.1% sevoflurane induced neurotoxicity in the neurons and H4 cells [14,21,22]. The neurons and the H4 cells were exposure to 4.1% sevoflurane in the carrier gas (95% air/5% CO2) for 15 min and the chamber was incubated at 37°C for 6 h as descried previously [14]. H4 cells were treated with Ac-YVAD-cmk (40 μM) [23] or Mitoquinone mesylate (MitoQ, 1μM) [24] 30 min before sevoflurane exposure. The neurons were administrated with Ac-YVAD-cmk (40 μM) 30 min before sevoflurane exposure.

### Immunoblot

The brain tissues and cells were homogenized in RIPA buffer (Beyotime, P0013B), with 1× protease inhibitor cocktail (Beyotime, P1010). Supernatants were collected by centrifuging at 16 200×g for 10 minutes, and the bicinchoninic acid protein assay kit was used to measure the protein concentration. (Beyotime, P0012S). Each sample was divided into an aliquot of 50g protein using SDS-PAGE, which was then transferred to nitrocellulose, which was blocked with 5% nonfat milk in PBS (pH 7.4). The membranes were incubated with primary antibodies against NLRP3 (1:1,000; ABclonal, A6564), Cleaved caspase-1 (1:1,000; ABclonal, A0964), IL-1β (1:500; Abclonal, A20527), IL-18 (1:1000; abcam, ab191860), LC3 (1:1,000; sigma, L7543), P62 (1:1,000; MBL, PM045), Tomm20 (1:1000; ABclonal, A19403), Cleaved Gasdermin D (1:1000; CST #36425), actin (1:5,000; ABclonal, AC026) 4°C overnight. Secondary antibodies conjugated with HRP against either rabbit or mouse IgG (1:5,000, CST, 7071 and 7072) were performed for 2 h at room temperature and blots were exposed to a chemiluminescent detection system using the SuperSignal West Pico Substrate (34077, Pierce) and exposed to film. Utilizing Quantity-One software (Bio-Rad), digital images were quantified using densitometric measurements.

### Immunostaining

The mice were anesthetized and perfused with 0.01 mol/L phosphate buffered saline (PBS, pH 7.2–7.4) followed by 4% paraformaldehyde in 0.1 mol/L PB through the ascending aorta. The brains were then removed, and post-fixed in the same solution for 2 h before cryoprotection in PB containing 15% sucrose at 4°C for 2 days, and the brains were removed into PB containing 30% sucrose at 4°C for another 2 days for dehydration. The brains were wrapped and embedded in aluminum foil with O.C.T and stored at -70°C until sectioning is performed. Every 25 μm thickness at hippocampus region, serially cut through the brain in cryostat, were collected.

Sections were sequentially incubated with the solution of 3% donkey serum, 0.3% Triton X-100 containing at room temperature for 1 h after antigen retrieval. Then the sections were incubated with antibody diluent containing goat antibody against cleaved caspase-1 (1:50; ABclonal, A0964), IL-1β (1:100; ABclonal, A20527) CD11b (1:50; Santa Cruz Biotechnology, sc-1186), caspase-1 p20 (1:80; Santa Cruz Biotechnology, sc-398715), NLRP3 (1:50, Invitrogen, 768319), HSP60 (1:50, ABclonal, A0969) and LAMP-2 (1:50; Santa Cruz Biotechnology, sc-20004), for 1 days at 4°C, then sections were rinsed with PBS (3×10 min) followed by Alexa Fluor™ 488 goat anti-mouse antibody, Alexa Fluor™ 594 goat anti-rabbit antibody, Goat anti-Rat IgG Cross-Adsorbed secondary antibody Alexa Fluor™ 594, and Goat anti-Rat IgG Cross-Adsorbed secondary antibody Alexa Fluor™ 488 for 1 h at room temperature. After rinsed with PBS (6×5 min), The signals were visualized under epifluorescence microscope and all the parameters used were kept consistent during capturing. Images were analyzed according to Image-Pro Plus 5.0 software. The Pearson’s correlation coefficient was calculated according to Image J software.

### Measurement of mitochondrial activities

A fluorometric assay kit (S0027) from Beyotime were utilized to quantify the ATP concentration and the assay was completed according to manufacturer’s instruction. The hippocampus was homogenized in lysis buffer. The supernatant was added to the substrate solution following centrifugation. Compared with BCA assay for protein quantitation, the calculation for concentration of ATP was according to the unit protein content (μmol/μg).

### Measurement of lipid peroxidation

The hippocampus was homogenized in IP lysis buffer. The supernatant was collected by centrifugation at 12 000×g for 10 min, and the protein concentration was tested by a bicinchoninic acid protein assay kit (Beyotime, P0012S). The Lipid Peroxidation malondialdehyde (MDA) assay kit was used to quantify the MDA concentration by (Beyotime, S0131). The calculation for concentration of MDA was according to the unit protein content (μm/mg).

### Detection of ROS production in situ

Dihydroethidium could be oxidized to red fluorescent molecule ethidium by superoxide. We used the DHE (Sigma-Aldrich) to detect the in situ ROS production in hippocampus. The brain sections were incubated with DHE and captured by fluorescent microscope all the parameters used were kept consistent during capturing. And the DHE fluorescence intensity was quantified according to ImageJ software.

### Mitochondrial reactive oxygen species

According to previous work [14], we measured regional mitochondrial ROS accumulation using the Mito-SOX reagent (M36008, Thermo Fisher, USA) and the intracellular ROS levels using the fluorescent probe dihydroethidium (DHE). Briefly, H4 cells and the neurons were treated with Ac-YVAD-cmk (40 μM) for 30 min in advance of 4.1% sevoflurane exposure for 6 h. Then 5 μM Mito-SOX working solution was applied for mitochondrial ROS accumulation. After incubating for 10 min at 37°C without light exposure treatment, excitation wavelengths were measured at 510 nm and emission at 580 nm by a fluorescence microplate reader (SpectraMax M5/M5e). After treatment with sevoflurane and Ac-YVAD-cmk, H4 cells and the neurons were incubated with 1 μM DHE (Bestbio, China) for 60 min at 37°C. Excitation wavelengths were measured at 518 nm and emission at 610 nm by a fluorescence microplate reader (SpectraMax M5/M5e).

### Mt-keima reporter assay

After seeding H4 cells for 16 h, mt-Keima reporters were transfected into H4 cells by Lipofectamine 3000 (Thermo Fisher, USA, L300015) and incubated for 8 h as described previously [14]. All experiments were performance after mt-Keima reporters transfection. H4 cells were treated with Ac-YVAD-cmk (40 μM) 30 min before 4.1% sevoflurane exposure. After sevoflurane treatment for 6 h, the cells were visualized under an epifluorescence microscope using 488 nm and 561 nm lasers immediately. All the parameters used were kept consistent during capturing. The 561-channel fluorescence signal indicated mitophagy flux.

### Transmission electron microscope

As described previously [14], the tissues from the hippocampus were fixed with 2.5% glutaraldehyde overnight at 4°C. The tissues were post-fixed with 1% osmium tetraoxide for 2 h, after rinsing with PBS. The tissues were then rinsed with distilled water, followed by a graded ethanol dehydration series ending with propylene oxide. After infiltration in a mixture of one-half propylene oxide and one-half resin, the tissues were embedded in resin. Sections (120 nm) were cut and stained with 4% uranylacetate for 20 min and with 0.5% lead citrate for 5 min. Hippocampal neurons were observed on a transmission electron microscope (TEM) (Phliphs Tecnai 10, Holland) in the Center of Cryo-Electron Microscopy at Zhejiang University.

### Statistical analysis

GraphPad Prism 8.0 software was used to process these data. All data were represented as mean ± standard deviation, and which were analyzed via one-way analysis of variance (ANOVA) and Tukey’s post hoc test. *P*<0.05 was considered as statistical significance.

## Results

### Sevoflurane enhanced the microglial inflammasome activation in aged mice

**Fig 1:**
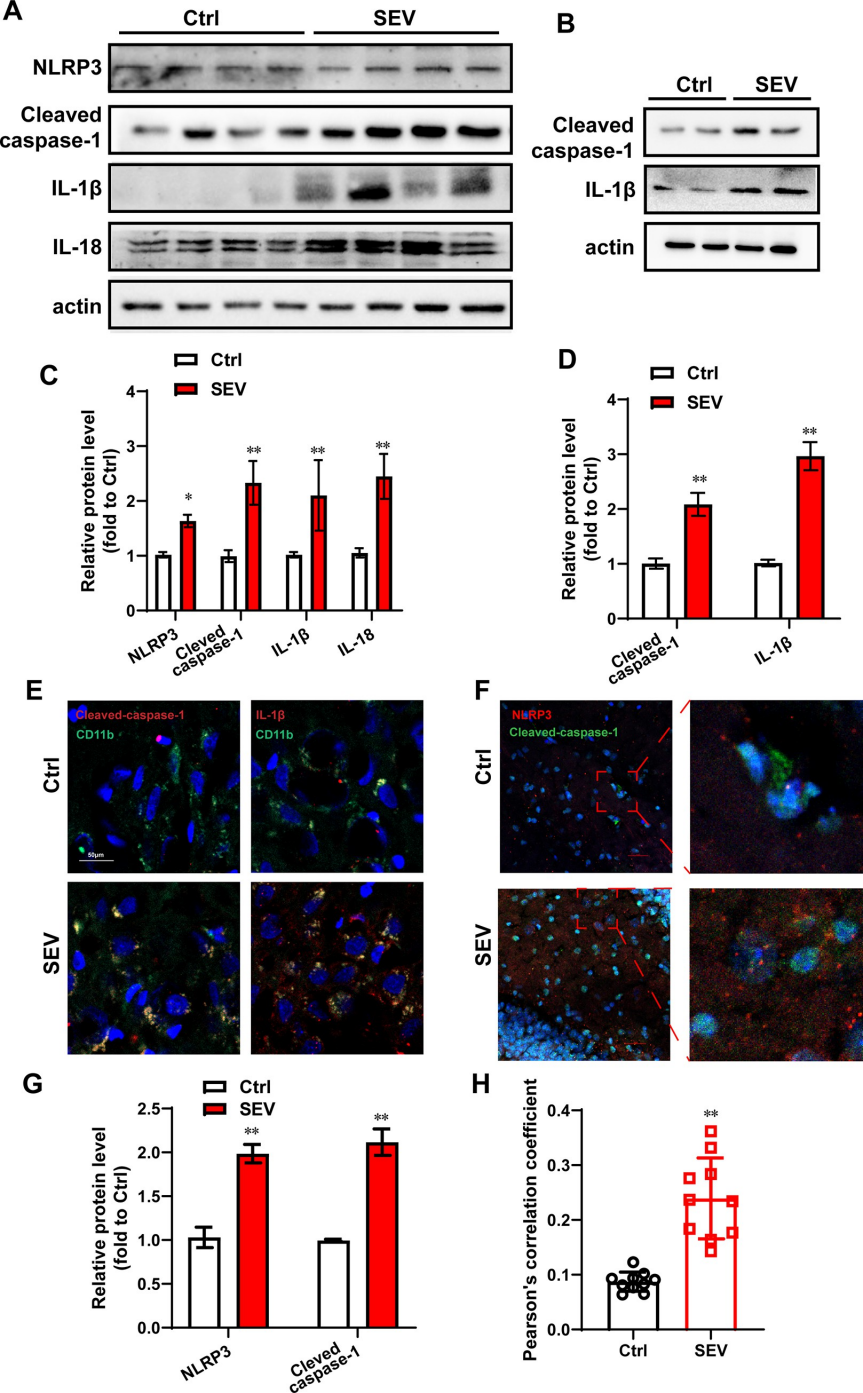
Sevoflurane enhanced the microglial inflammasome activation in aged mice. The 18-month-old mice were treatment with 2% sevoflurane for 5 h. The hippocampus of all the aged mice were harvested. (A) Comparison of NLRP3, Cleaved caspas-1, IL-1β and IL-18 expression in the hippocampus of aged mice in each group. β-actin was used as an endogenous control; (C) The semi-quantitative analysis for the blotting; n = 4; (B) The primary hippocampal neurons were treated with 4.1% sevoflurane for 6 h. The Cleaved caspase-1 and IL-1β protein levels were determined by western blotting; (D) The results of semi-quantitative analysis of Cleaved caspase-1 and IL-1β are shown. The experiment was repeated for three times; (E) The expression of CD11b and cleaved-caspase-1, CD11b and IL-1β in the hippocampus were measured by immunofluorescence assay. n = 3. Scale bar represents 50 μm; (F) The expression of NLRP3 and cleaved caspase-1 in the hippocampus were measured by immunofluorescence assay. n = 3. Scale bar represents 25 μm. (G) The semi-quantitative analysis for the immunofluorescence images of NLRP3 and cleaved caspase-1. (H) The Pearson’s correlation coefficient was shown. The data are expressed as mean ± SD. * *P*<0.05, **P<0.01, Ctrl vs SEV.

The 18-month-old mice were treatment with 2% sevoflurane for 5 h. To explore the impact of NLRP3 inflammasome activation on aged mice after sevoflurane treatment, cleaved caspase-1, IL-1β and IL-18 maturation were measured by western blotting (Fig 1A). According to the semi-quantitative analysis, sevoflurane enhanced the expression level of NLRP3, cleaved caspase-1, IL-1β and IL-18 (Fig 1C). The primary hippocampal neuron was exposed by 4.1% sevoflurane for 6 h. The protein levels of cleaved caspase-1 and IL-1β were detected in primary hippocampal neuron after sevoflurane treatment by western blotting (Fig 1B). The semi-quantitative analysis suggested that sevoflurane increased the expression level of cleaved caspase-1 and IL-1β in primary hippocampal neuron (Fig 1D). Further, to explore the special localization of inflammasomes in nerve cells after sevoflurane treatment in aged mice, the immunostaining was measured in hippocampus of aged mice (Fig 1E). The results indicated that sevoflurane increased the level of cleaved caspase-1 and IL-1β in CA3 of aged mice and the NLRP3 inflammasomes activator proteins immune-colocalized with microglia activator protein (CD11b) (S1 Fig).

We measured the colocalization of NLRP3 and Cleaved caspase-1 in hippocampus of aged mice after sevoflurane treatment by immunostaining assay. Sevoflurane increased the expression level of NLRP3 and Cleaved caspase-1 in hippocampus of aged mice. (Fig 1F and 1G) Moreover, we used Pearson’s correlation coefficient (PCC) to quantify colocalization of NLRP3 and Cleaved caspase-1. The PCC is a well-established measure of correlation has range of +1 (perfect correlation) to −1 (perfect but negative correlation) with 0 denoting the absence of a relationship [25]. In our work, the mean PCC in control group is 0.0873. Sevoflurane increased the mean PPC to 0.2393 significantly (*p*<0.001). (Fig 1H) The above data suggested in hippocampus of aged mice after sevoflurane treatment, cleaved caspase-1 was activated by the high level of NLRP3.

### Ac-YVAD-cmk reduced the inflammasome activation induced by sevoflurane

**Fig 2:**
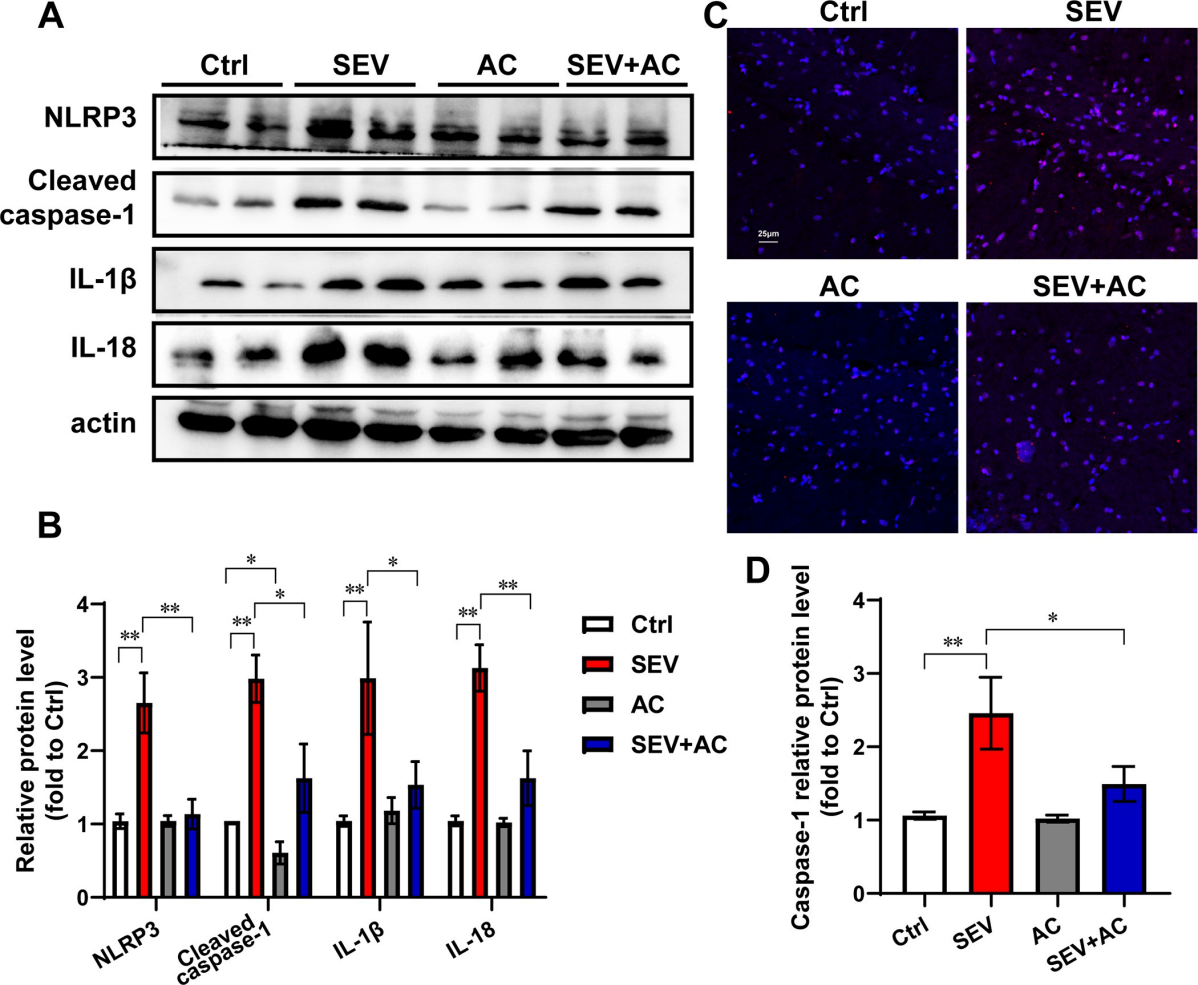
Ac-YVAD-cmk reduced the inflammasome activation induced by sevoflurane. The aged-mice were given the caspse-1 inhibitor Ac-YVAD-cmk (12.5 μmol/kg, i.p.) before sevoflurane treatment. The hippocampus of all the aged mice were harvested. (A) Comparison of NLRP3, Cleaved caspas-1, IL-1β and IL-18 expression in the hippocampus of aged mice in each group. β-actin was used as an endogenous control. (B) The semi-quantitative analysis for the blotting. n = 6; (C) The expression of cleaved-caspase-1 in the hippocampus were measured by immunofluorescence assay. Scale bar represents 50 μm. n = 3; (D) The semi-quantitative analysis for the immunofluorescence images. The data are expressed as mean ± SD. * *P*<0.05, **P<0.01.

In order to determine the inflammasome activation in aged sevoflurane mice after sevoflurane treatment, the mice were treatment with the caspse-1 inhibitor Ac-YVAD-cmk before sevoflurane treatment. The inflammasome activation in hippocampus of aged mice were detected by western blotting. The semi-quantitative analysis indicated Ac-YVAD-cmk reduced the high expression level of NLRP3, cleaved caspase-1, IL-1β and IL-18 in aged mice after sevoflurane treatment. (Fig 2A and 2B) In immunostaining assay, Ac-YVAD-cmk decreased the upregulated level of cleaved caspase-1 induced by sevoflurane in CA3. (Fig 2C and 2D) Ac-YVAD-cmk decreased the upregulated level of NLRP3 and cleaved caspase-1 induced by sevoflurane in primary hippocampus neurons. (S3A and S3B Fig). The above results suggested Ac-YVAD-cmk reduced the inflammasome activation induced by sevoflurane, and inflammasome activation may contribute to sevoflurane-induced neurotoxicity.

### Ac-YVAD-cmk rescued learning ability impairment induced by sevoflurane in aged mice

**Fig 3:**
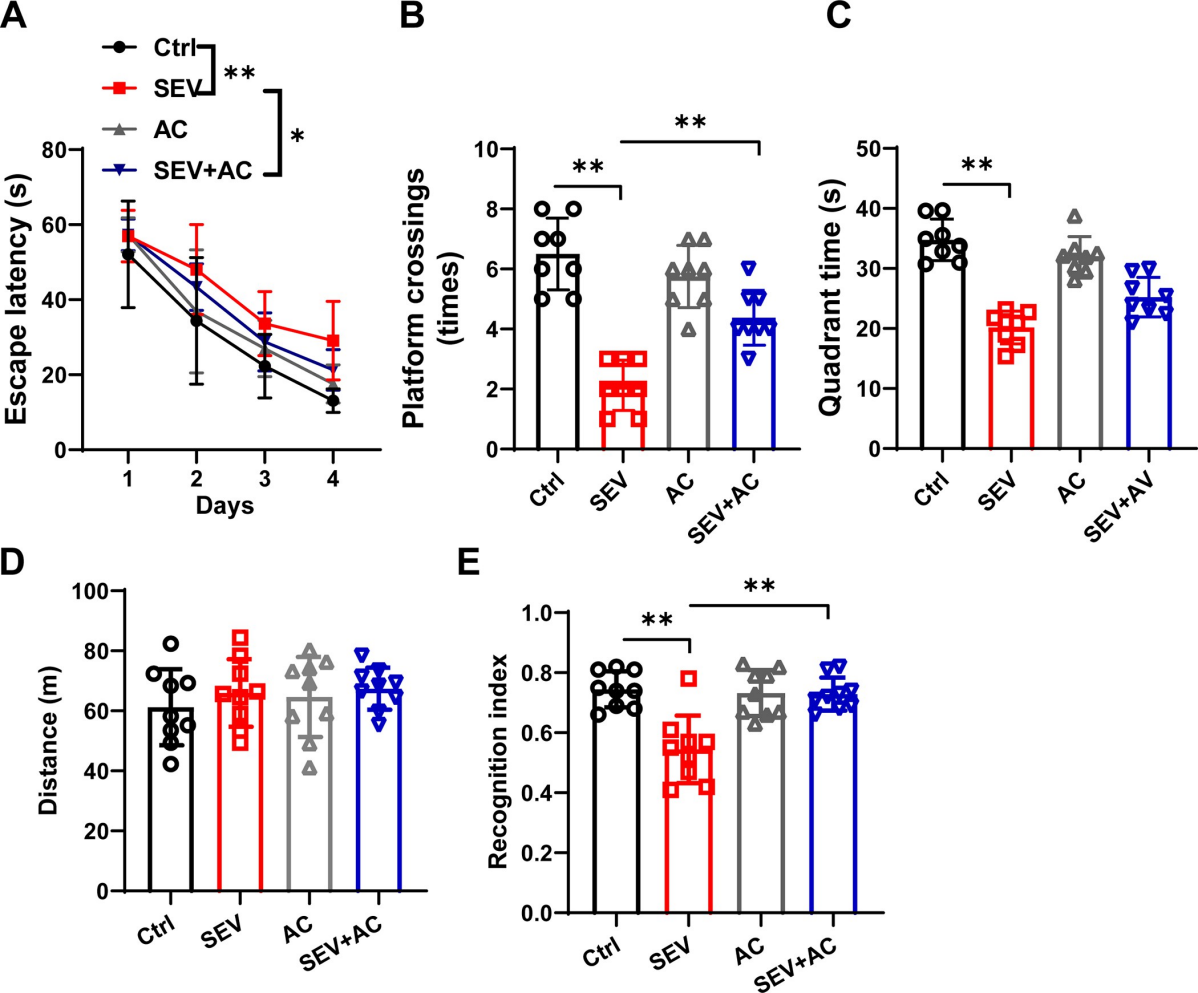
Ac-YVAD-cmk rescued learning ability impairment induced by sevoflurane in aged mice. Eighteen-month-old mice were subjected to 2% sevoflurane for 5 h. Ac-YVAD-cmk (12.5 μmol/kg) was administrated intraperitoneally 1 h before sevoflurane treatment. The Morris Water Maze was used to test the learning ability. The parameters escape latency (A) number of platform crossings (B) and quadrant time (C) were measured. n = 8. The novel object recognition test was performance. The travelled distance (D) and recognition index (E) were shown. n = 9.The data are expressed as mean ± SD. * *P*<0.05, ** *P*<0.01.

Several researches reported that the neurotoxicity of sevoflurane induced learning impairment [14,26]. To explore the effect of Ac-YVAD-cmk in learning ability, the spatial learning ability of aged mice were measured by Morris water maze 24 h after the sevoflurane with or without Ac-YVAD-cmk treatment. The escape latency (Fig 3A) was longer in SEV group than that in control group. The platform crossings (Fig 3B) and quadrant time (Fig 3C) were decreased in sevoflurane mice compared with the control mice. Ac-YVAD-cmk decreased the escape latency and increased the platform crossings in the aged mice after sevoflurane treatment. (Fig 3A and 3B).

We also used the novel objective recognition test 24 h after the sevoflurane with or without Ac-YVAD-cmk treatment. The overall distance of traveling was not significant difference (Fig 3D), suggesting sevoflurane did not cause motor function impairment. The recognition index was decreased in the SEV group compared to the Ctrl group, and Ac-YVAD-cmk rescued the decreased recognition index after sevoflurane treatment. (Fig 3E) The above data indicated that Ac-YVAD-cmk rescued learning ability impairment induced by sevoflurane in aged mice.

### Ac-YVAD-cmk mitigated mitochondria dysfunction induced by sevoflurane

**Fig 4:**
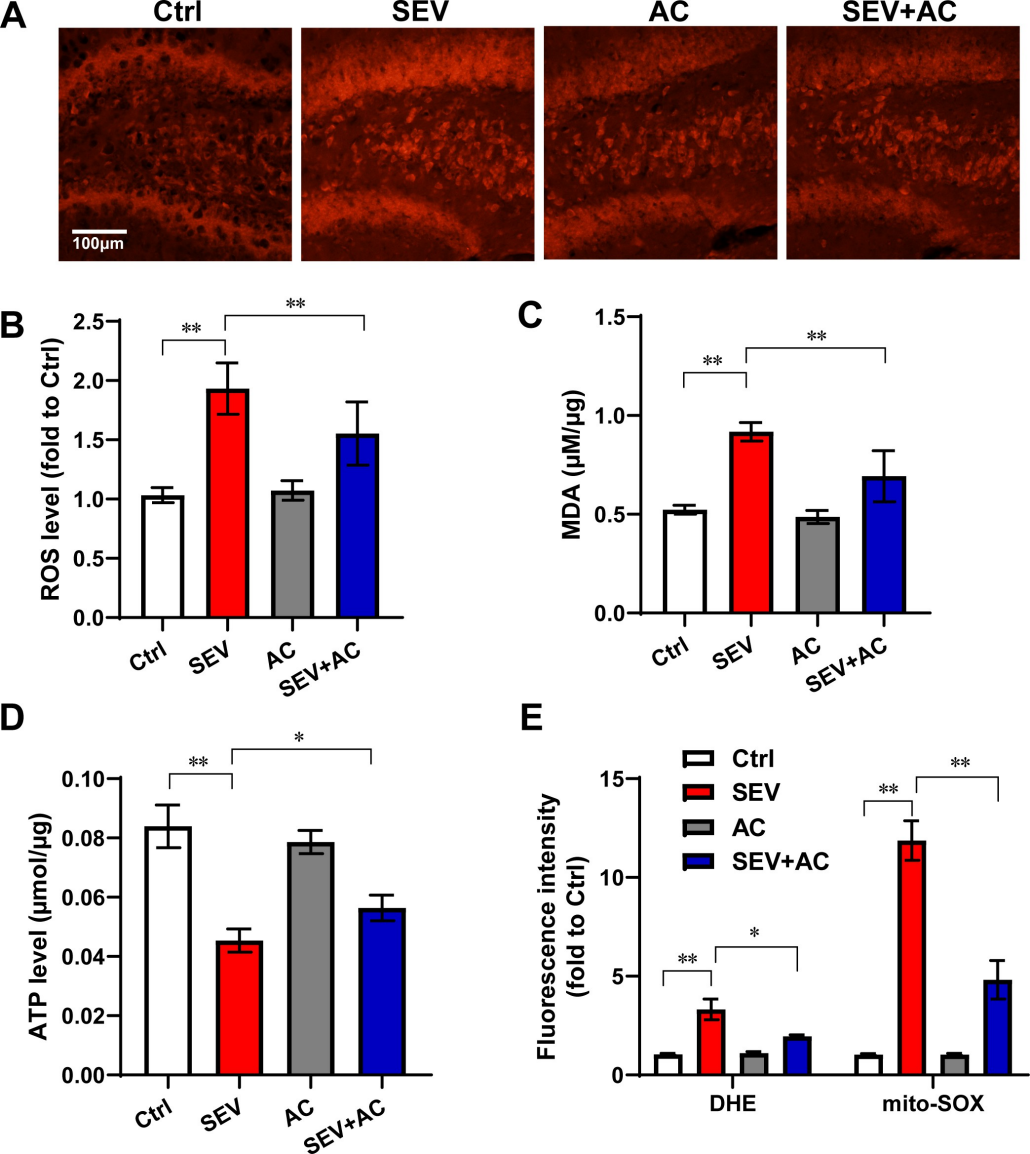
Ac-YVAD-cmk mitigated mitochondria dysfunction induced by sevoflurane. Eighteen-month-old mice were subjected to 2% sevoflurane for 5 h. Ac-YVAD-cmk (12.5 μmol/kg) was administrated intraperitoneally before sevoflurane treatment. (A) DHE staining in hippocampus. Scale bar represents 100 μm. (B) The semi-quantitative analysis for the DHE staining. n = 3. (C) The MDA assay of hippocampus. n = 4; (D) The ATP level of hippocampus. n = 4; (D) H4 cells were treated with Ac-YVAD-cmk (40 μM) for 30 min in advance of 4.1% sevoflurane exposure for 6 h. The intracellular ROS and mitochondrial ROS levels were measured. n = 6. The data are expressed as mean ± SD. * *P*<0.05, **P<0.01.

Our previous work reported that mitophagy dysfunction was one of the mechanisms underlying sevoflurane-induced learning impairment [14]. In order to explore the relationship between NLRP3 inflammasomes and mitochondria function underlying learning ability impairment induced by sevoflurane, the mitochondria function was measured. The generation of reactive oxygen species (ROS) was detected qualitatively in fresh frozen hippocampal sections by DHE staining. Sevoflurane increased the ROS fluorescence level in hippocampal sections of aged mice. And the hippocampus from the sevoflurane and Ac-YVAD-cmk group showed weaker DHE staining compared with the sevoflurane mice. (Fig 4A and 4B)

In MAD assay, Ac-YVAD-cmk revised the high level of lipid peroxidation induced by sevoflurane in aged mice (Fig 4C). Moreover, we found the aged mice after sevoflurane treatment exhibited lower baseline ATP concentrations in the hippocampus as compared with the control aged mice. Ac-YVAD-cmk increased ATP concentrations in hippocampal mice of aged mice as compared with sevoflurane mice (Fig 4D).

In order to test the effect of Ac-YVAD-cmk on mitochondrial ROS, we measured regional mitochondrial ROS accumulation using the Mito-SOX reagent and the intracellular ROS levels using the fluorescent probe dihydroethidium in H4 cells. H4 cells were treated with Ac-YVAD-cmk (40 μM) 30 min before 4.1% sevoflurane exposure. The data indicated that sevoflurane increased the intracellular ROS level by 3.3-fold and the mitochondrial ROS level by 11.8-fold. And Ac-YVAD-cmk relieved the intracellular ROS level and mitochondrial ROS accumulation induced by sevoflurane (Fig 4E). Ac-YVAD-cmk also decreased the mitochondrial ROS level significantly when the primary hippocampus neurons were exposure to sevoflurane. (S3C Fig) The above results suggested Ac-YVAD-cmk mitigated the sevoflurane-induced mitochondria dysfunction.

### Ac-YVAD-cmk reversed the mitophagy impairment induced by sevoflurane

**Fig 5:**
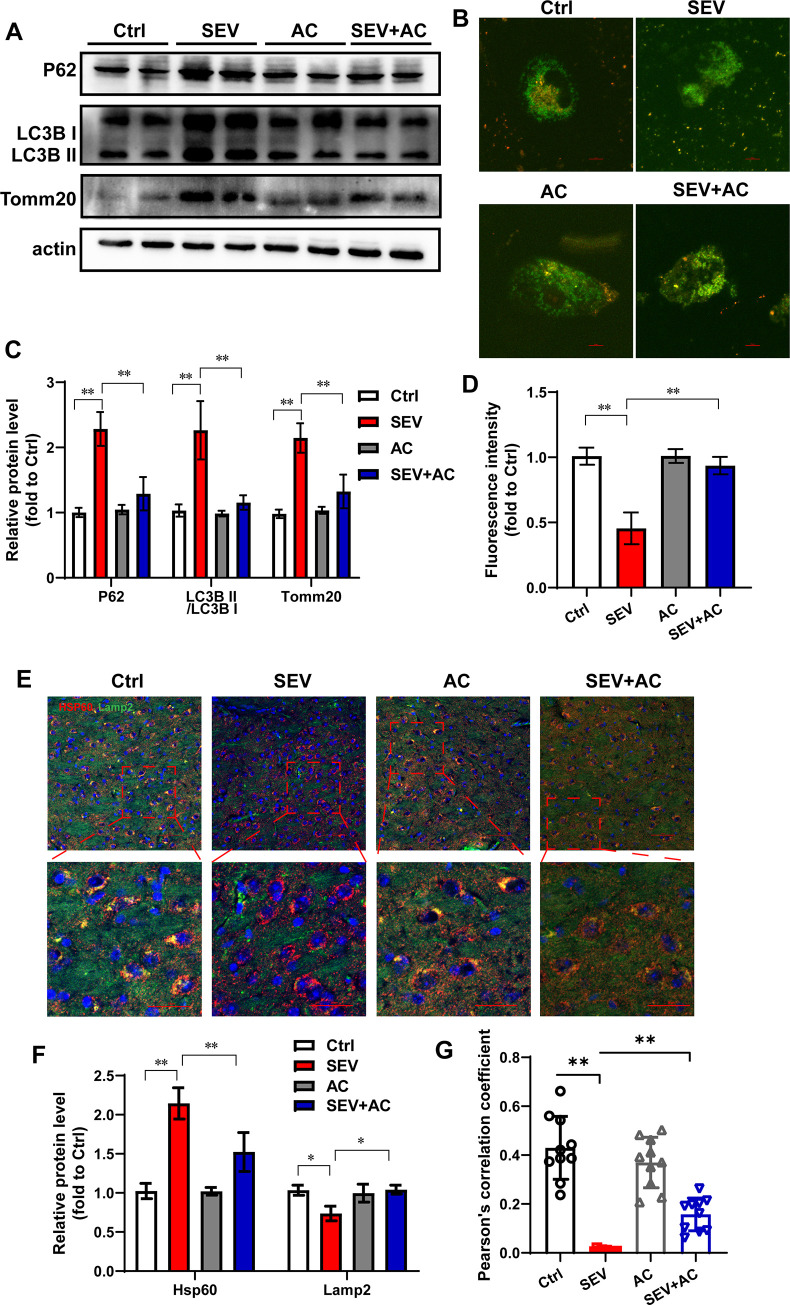
Ac-YVAD-cmk reversed the mitophagy impairment induced by sevoflurane. Eighteen-month-old mice were subjected to 2% sevoflurane for 5 h. Ac-YVAD-cmk (12.5 μmol/kg) was administrated intraperitoneally before sevoflurane treatment. (A) Comparison of p62, LC3B, and Tomm20 expression in the hippocampus of aged mice in each group. β-actin was used as an endogenous control. (C) The semi-quantitative analysis for the blotting. n = 5; (B) After mt-Keima reporters transfected, H4 cells were treated with Ac-YVAD-cmk (40 μM) 30 min before 4.1% sevoflurane exposure. Images show representative examples from three independent experiments for each group. (D) The 589-channel fluorescence were analysis. n = 6. (E) The expression of LAMP2 and HSP60 in the hippocampus were measured by immunofluorescence assay. Scale bar represents 50 μm and 25 μm in enlarged images. n = 3. (F) The semi-quantitative analysis for the immunofluorescence images of LAMP2 and HSP60. (G) The Pearson’s correlation coefficient was shown. The data are expressed as mean ± SD. * *P*<0.05, **P<0.01.

Mitophagy is the removal of damaged mitochondria through autophagy to regulate mitochondrial quality. In order to effect of Ac-YVAD-cmk on mitophagy flux in hippocampus of aged mice after sevoflurane, western blotting was used. Sevoflurane enhanced the LC3B II/I and accumulated the level of P62, and increased the expression level of Tomm20. (Fig 5A and 5C) The results suggested that sevoflurane inhibited mitophagy in hippocampus. And in the sevoflurane mice, Ac-YVAD-cmk blocked the high level of LC3B II/I, P62 and Tomm20, reversed the mitophagy flux to normal level.

In order to detect the mitophagy flux, the H4 cells were transfected with mt-Keima reporters. Administration of the mitophagy inducer CCCP significantly increased red fluorescence in H4 cells, suggesting that mitophagy was induced (S4C Fig). H4 cells were treated with sevoflurane and Ac-YVAD-cmk. H4 cells treated with sevoflurane had lower red fluorescence compared with control cells, indicating sevoflurane caused mitophagy dysfunction. Ac-YVAD-cmk rescued the mitophagy flux by increasing the red fluorescence. These data suggested that Ac-YVAD-cmk alleviated the mitophagy impairment induced by sevoflurane.

We measured the colocalization of a mitochondrial matrix protein, HSP60 and lysosomal membrane protein, LAMP2 in hippocampus of aged mice after sevoflurane and Ac-YVAD-cmk treatment by immunostaining assay. The expression level of HSP60 were higher in the sevoflurane mice than that of control mice, Ac-YVAD-cmk decreased the high level of HSP60 after sevoflurane treatment. Sevoflurane reduced the expression level of LAMP2 in hippocampus. In the SEV+AC group, the expression level of LAMP2 were higher than that of in the SEV group. (Fig 5E and 5F) Moreover, we used Pearson’s correlation coefficient (PCC) to quantify colocalization of HSP60 and LAMP2. Ac-YVAD-cmk increased the PPC which was reduced by sevoflurane. (Fig 5G) The above results indicated sevoflurane promoted mitochondria accumulation and lysosome dysfunction resulting mitophagic flux inhibition and further verified the positive role of Ac-YVAD-cmk on the mitophagy flux dysfunction induced by sevoflurane.

## Discussion

The underlying pathogenesis of POCD is still unknown. Our previous work revealed the role of mitophagy on the cognitive impairment in aged mice after treatment with sevoflurane [14]. In present work, we provided evidence that NLRP3 inflammasomes was involved in postoperative cognitive impairment. Utilization of caspase-1 inhibitor, Ac-YVAD-cmk mitigated sevoflurane-induced learning ability impairment. Moreover, we found that for the first time Ac-YVAD-cmk mitigated mitochondria function and revised mitophagy flux induced by sevoflurane. The current work disclosed Ac-YVAD-cmk treatments for POCD are beneficial for the improvement outcome, and provide a proof involving the interaction between inflammasomes and mitophagy.

Dysregulated activation of inflammasomes, mitophagy dysfunction are relevant to pathogenesis of neurodegenerative diseases [27,28]. Disturbance of the mitochondria following mitochondria ROS generation triggered inflammasome activation [29]. Meanwhile, mitophagy is required for clearance the damaged mitochondria [30]. Mitophagy dysfunction accumulated the production of mitochondrial ROS and increased the translocation of mitochondria DNA into the cytosol. The above risks intensified the inflammasome activation [31,32]. A series of reports revealed ameliorated cognitive dysfunction through the activated of mitophagy and subsequently inhibition of the NLRP3 inflammasome [10,27,33]. These reports suggested that mitophagy dysfunction regulated inflammasome activation. In addition, inflammasome activation leads to caspase-1 dependent mitochondrial damage regulating mitophagy flux. The recent research suggested inflammasomes mediated Parkin cleavage inhibiting mitophagy [34]. The danger-associated molecular patterns (DAMP) triggers mitochondrial outer membrane permeabilization and mitochondrial permeability. And inflammasome activation might leads to block of mitophagy. In present study, in the aged mice following sevoflurane treatment, we found Ac-YVAD-cmk decreased the expression of cleaved caspase-1, IL-1β and IL-18 inhibiting the activation of NLRP3 (Fig 2). As a distinguished inhibitor of caspase-1, Ac-YVAD-cmk mitigated the sevoflurane-induced mitochondria dysfunction through reducing the lipid peroxidation and mitochondrial ROS accumulation (Fig 3). Moreover, our work suggested, for the first time, that Ac-YVAD-cmk blocked the high level of LC3B II/I, P62 and Tomm20 induced by sevoflurane in aged mice and revising the mitophagy flux by using mt-Keima reporters in H4 cells. Ac-YVAD-cmk increased the colocalization with HSP60 and LAMP2 in hippocampus which was reduced by sevoflurane. We found for the first time Ac-YVAD-cmk ameliorated sevoflurane-induced learning ability impairment through NLRP3 inflammasomes activation subsequent revising mitophagy flux.

A couple of reports suggested that NLRP3 inflammasome regulated autophagy. NLRP3 was reported to interact with Beclin 1, a protein promoted in autophagy initiation, through NACHT domain [35]. Our previous work indicated sevoflurane induced mitophagy dysfunction through inactivation of Parkin expression. Overexpression of PARK2, but not mutant PARK2 lacking enzyme activity, in neuron cell lines declined ROS and Tomm20 accumulation and reversed mitophagy dysfunction following sevoflurane treatment [14]. In bone marrow-derived macrophages, inflammasome activation caspase-1-mediated cleavage of Parkin is required to caspase-1-dependent inhibition of mitophagy [34]. In the present work, Ac-YVAD-cmk might mediate cleavage of Parkin through the caspase-1 activation resulting revising mitophagy impairment.

Ac-YVAD-cmk, a well-known selective inhibitor of caspase-1, has been reported to exert a protective effect on the brain, liver, and lung in mouse models of septic shock in previous studies [4,29,36]. Ac-YVAD-cmk decreased the release of mature IL-1β/IL-18 and microglia activation in perihematomal tissue of ICH rat improving the behavioral performance [23]. Ac-YVAD-cmk pretreatment attenuated isoflurane-induced NLRP-caspase-1 pathway and learning ability impairment [4]. In the current study, we found Ac-YVAD-cmk ameliorated NLRP3 inflammasome activation induced by sevoflurane, revised mitophagy flux, improved cognitive performance. Pretreatment of Ac-YVAD-cmk is regarded as a novel therapeutic strategy for neuroinflammation-induced learning ability impairment.

The E17 mice hippocampal neurons were widely used in neurobiological research. Some research teams utilized primary hippocampal neurons to explore the neurotoxicity of sevoflurane [22,37,38]. Primary neurons receive and transmit chemical or electric signals and directly take part in the signal transduction. In neurons, synaptic plasticity is one of the important neurochemical foundations of learning and memory [39]. However, it’s not easy to transfect primary neurons with chemical reagents [40]. The low transfection efficiency limits the application of primary neurons. In our current study, the results of western blots in primary hippocampus neurons suggested that NLRP3 inflammasomes activation may be involved in cognition dysfunction induced by sevoflurane, and Ac-YVAD-cmk reduced the inflammasome activation induced by sevoflurane. We also measured regional mitochondrial ROS accumulation and the intracellular ROS levels in primary hippocampal neuron. Ac-YVAD-cmk relieved the mitochondrial ROS accumulation induced by sevoflurane. These results suggested Ac-YVAD-cmk alleviated the sevoflurane-induced cognition dysfunction, partly due to mitigation of mitochondria damage. The glial cells perform a role in regulating homeostasis of the nervous system which provides adequate protection and support in the functioning of the nervous system [41]. H4 human neuroglioma cells have been utilized as an alternative in several previous studies [14,21,42], despite their immortal nature. High transfection efficiency in H4 cells help us to explore many complex physiological and pathological mechanisms in nervous system. In order to detect the mitophagy flux, mt-Keima reporters were transfected into H4 cells by Lipofectamine 3000. Ac-YVAD-cmk rescued the mitophagy flux by increasing the red fluorescence. We utilized H4 cells to further verify the positive role of Ac-YVAD-cmk on the mitophagy flux dysfunction induced by sevoflurane, resulting relieved cognitive impairment. According to the characteristic of primary hippocampus neuron and H4 human neuroglioma cells, we fully utilized these two types of nerve cells to explore the mechanisms of cognitive impairment in aged mice induced by sevoflurane. We verified that Ac-YVAD-cmk ameliorated sevoflurane-induced cognitive dysfunction and revised mitophagy impairment.

As a form of cell death, pyroptosis is characterized by the activation of NLRP3 inflammasomes, the formation of cell membrane pore and mediated by gasdermin (GSDM) family protein (GSDMD and GSDME) [43,44]. Proinflammatory Caspases (Casps) induce cleaving the protein GSDMD, which targets cellular membranes and leads to plasma membrane permeabilization [45]. In our study, we found sevoflurane induced the expression of Cleaved GSDMD in hippocampus of aged mice (S5A and S5B Fig). Moreover, the outer layer of the nuclear membrane was compromised by sevoflurane, as the ultrastructural damages of cells in hippocampus shown by TEM. The rupture of the plasma membrane in extremis led to the extravasation of cellular contents, as shown by the reduced abundance of intracellular dense particles (S5C Fig). The above results suggested that pyroptosis was involved in the cognitive impairment induced by sevoflurane. Some specific inhibitor of pyroptosis, like necrosulfonamide should be used to clarify the role of pyroptosis in cognitive impairment further.

In the current study, we identified NLRP3 inflammasomes activation as a cause of sevoflurane-induced cognitive impairment. We promote that, for the first time, Ac-YVAD-cmk ameliorated NLRP3 inflammasome activation induced by sevoflurane and revised mitophagy flux.

## Supporting information

S1 Fig (A) The results of semi-quantitative analysis of Cleaved caspase-1 and CD11b are shown. (B) The results of semi-quantitative analysis of IL-1β and CD11b are shown. The data are expressed as mean ± SD. * *P*<0.05, **P<0.01, Ctrl vs SEV. The experiment was repeated for three times.
(TIF)

S2 Fig MCC950 rescued learning ability impairment induced by sevoflurane in aged mice. Eighteen-month-old mice were subjected to 2% sevoflurane for 5 h. aged mice were injected intraperitoneally (i.p.) with 50 mg/kg MCC950 or vehicle control (DMSO/PBS) 1 h h before sevoflurane treatment. The Morris Water Maze was used to test the learning ability. The parameters escape latency (A) number of platform crossings (B) and quadrant time (C) were measured. n = 8. The data are expressed as mean ± SD. * *P*<0.05, **P<0.01.
(TIF)

S3 Fig The primary hippocampus neurons were treated with Ac-YVAD-cmk (40 μM) 30 min before sevoflurane exposure. (A) Comparison of NLRP3 and Cleaved caspas-1 expression in each group. β-actin was used as an endogenous control. (B) The semi-quantitative analysis for the blotting. n = 6; (C) The intracellular ROS and mitochondrial ROS levels were measured. n = 9. The data are expressed as mean ± SD. * *P*<0.05, **P<0.01.
(TIF)

S4 Fig H4 cells were treated with Mitoquinone mesylate (MitoQ, 1μM) 30 min before sevoflurane exposure. (A) Comparison of NLRP3 and Cleaved caspas-1 expression in each group. β-actin was used as an endogenous control. (B) The semi-quantitative analysis for the blotting. n = 6; (C) The more images of the mito-Keima in Fig 5B were shown. The image of the mito-Keima after CCCP treatment was also shown as positive control. The data are expressed as mean ± SD. * *P*<0.05, **P<0.01.
(TIF)

S5 Fig The 18-month-old mice were treatment with 2% sevoflurane for 5 h. The hippocampus of all the aged mice were harvested. (A) Comparison of Cleaved GSDMD expression in the hippocampus of aged mice in each group. β-actin was used as an endogenous control; (B) The semi-quantitative analysis for the blotting; n = 6; (C) the ultrastructural damages of cells in hippocampus. n = 3; The data are expressed as mean ± SD. **P<0.01.
(TIF)

S1 Raw images (ZIP)

S2 Raw images (ZIP)

S3 Raw images (ZIP)

S4 Raw images (ZIP)

S5 Raw images (ZIP)

S6 Raw images (ZIP)

S7 Raw images (ZIP)

## References

[1] Bedford PD. Adverse cerebral effects of anaesthesia on old people. Lancet 1955; 269 (6884): 259–63. doi: 10.1016/s0140-6736(55)92689-1 13243706

[2] Huang JM, Lv ZT, Zhang B, Jiang WX, Nie MB. Intravenous parecoxib for early postoperative cognitive dysfunction in elderly patients: evidence from a meta-analysis. Expert review of clinical pharmacology 2020; 13 (4): 451–60. doi: 10.1080/17512433.2020.1732815 32077347

[3] Wang CM, Chen WC, Zhang Y, Lin S, He HF. Update on the Mechanism and Treatment of Sevoflurane-Induced Postoperative Cognitive Dysfunction. Frontiers in aging neuroscience 2021; 13: 702231. doi: 10.3389/fnagi.2021.702231 34305576 PMC8296910

[4] Wang Z, Meng S, Cao L, Chen Y, Zuo Z, Peng S. Critical role of NLRP3-caspase-1 pathway in age-dependent isoflurane-induced microglial inflammatory response and cognitive impairment. Journal of neuroinflammation 2018; 15 (1): 109. doi: 10.1186/s12974-018-1137-1 29665808 PMC5904978

[5] Zhang X, Zhou Y, Xu M, Chen G. Autophagy Is Involved in the Sevoflurane Anesthesia-Induced Cognitive Dysfunction of Aged Rats. PloS one 2016; 11 (4): e0153505. doi: 10.1371/journal.pone.0153505 27111854 PMC4844142

[6] Xu Z, Qian B. Sevoflurane anesthesia-mediated oxidative stress and cognitive impairment in hippocampal neurons of old rats can be ameliorated by expression of brain derived neurotrophic factor. Neuroscience letters 2020; 721: 134785. doi: 10.1016/j.neulet.2020.134785 32027953

[7] Hu N, Guo D, Wang H, Xie K, Wang C, Li Y, et al. Involvement of the blood-brain barrier opening in cognitive decline in aged rats following orthopedic surgery and high concentration of sevoflurane inhalation. Brain research 2014; 1551: 13–24. doi: 10.1016/j.brainres.2014.01.015 24440777

[8] Shen Y, Zhou T, Liu X, Liu Y, Li Y, Zeng D, et al. Sevoflurane-Induced miR-211-5p Promotes Neuronal Apoptosis by Inhibiting Efemp2. ASN neuro 2021; 13: 17590914211035036. doi: 10.1177/17590914211035036 34730432 PMC8819752

[9] Heneka MT, Kummer MP, Stutz A, Delekate A, Schwartz S, Vieira-Saecker A, et al. NLRP3 is activated in Alzheimer’s disease and contributes to pathology in APP/PS1 mice. Nature 2013; 493 (7434): 674–8. doi: 10.1038/nature11729 23254930 PMC3812809

[10] Ye JS, Chen L, Lu YY, Lei SQ, Peng M, Xia ZY. Honokiol-Mediated Mitophagy Ameliorates Postoperative Cognitive Impairment Induced by Surgery/Sevoflurane via Inhibiting the Activation of NLRP3 Inflammasome in the Hippocampus. Oxidative medicine and cellular longevity 2019; 2019: 8639618. doi: 10.1155/2019/8639618 30918581 PMC6409065

[11] Ising C, Venegas C, Zhang S, Scheiblich H, Schmidt SV, Vieira-Saecker A, et al. NLRP3 inflammasome activation drives tau pathology. Nature 2019; 575 (7784): 669–73. doi: 10.1038/s41586-019-1769-z 31748742 PMC7324015

[12] Kerr JS, Adriaanse BA, Greig NH, Mattson MP, Cader MZ, Bohr VA, et al. Mitophagy and Alzheimer’s Disease: Cellular and Molecular Mechanisms. Trends in neurosciences 2017; 40 (3): 151–66. doi: 10.1016/j.tins.2017.01.002 28190529 PMC5341618

[13] Killackey SA, Philpott DJ, Girardin SE. Mitophagy pathways in health and disease. The Journal of cell biology 2020; 219 (11). doi: 10.1083/jcb.202004029 32926082 PMC7594502

[14] Chen Y, Zhang P, Lin X, Zhang H, Miao J, Zhou Y, et al. Mitophagy impairment is involved in sevoflurane-induced cognitive dysfunction in aged rats. Aging 2020; 12 (17): 17235–56. doi: 10.18632/aging.103673 32903215 PMC7521530

[15] Zhou R, Yazdi AS, Menu P, Tschopp J. A role for mitochondria in NLRP3 inflammasome activation. Nature 2011; 469 (7329): 221–5. doi: 10.1038/nature09663 21124315

[16] Zhang F, Wang L, Wang JJ, Luo PF, Wang XT, Xia ZF. The caspase-1 inhibitor AC-YVAD-CMK attenuates acute gastric injury in mice: involvement of silencing NLRP3 inflammasome activities. Scientific reports 2016; 6: 24166. doi: 10.1038/srep24166 27053298 PMC4823746

[17] Coll RC, Robertson AA, Chae JJ, Higgins SC, Munoz-Planillo R, Inserra MC, et al. A small-molecule inhibitor of the NLRP3 inflammasome for the treatment of inflammatory diseases. Nature medicine 2015; 21 (3): 248–55. doi: 10.1038/nm.3806 25686105 PMC4392179

[18] Chen YR, Zhang SX, Fang M, Zhang P, Zhou YF, Yu X, et al. Egr2 contributes to age-dependent vulnerability to sevoflurane-induced cognitive deficits in mice. Acta pharmacologica Sinica 2022. doi: 10.1038/s41401-022-00915-5 35577909 PMC9622904

[19] Zhang P, Chen Y, Zhang S, Chen G. Mitochondria-Related Ferroptosis Drives Cognitive Deficits in Neonatal Mice Following Sevoflurane Administration. Frontiers in medicine 2022; 9: 887062. doi: 10.3389/fmed.2022.887062 35935755 PMC9355652

[20] Zhang CH, Fan YY, Wang XF, Xiong JY, Tang YY, Gao JQ, et al. Acidic preconditioning protects against ischemia-induced brain injury. Neuroscience letters 2012; 523 (1): 3–8. doi: 10.1016/j.neulet.2012.05.015 22583767

[21] Zhou YF, Wang QX, Zhou HY, Chen G. Autophagy activation prevents sevoflurane-induced neurotoxicity in H4 human neuroglioma cells. Acta pharmacologica Sinica 2016; 37 (5): 580–8. doi: 10.1038/aps.2016.6 27041458 PMC4857550

[22] Zhang J, Dong Y, Zhou C, Zhang Y, Xie Z. Anesthetic sevoflurane reduces levels of hippocalcin and postsynaptic density protein 95. Molecular neurobiology 2015; 51 (3): 853–63. doi: 10.1007/s12035-014-8746-1 24870966

[23] Liang H, Sun Y, Gao A, Zhang N, Jia Y, Yang S, et al. Ac-YVAD-cmk improves neurological function by inhibiting caspase-1-mediated inflammatory response in the intracerebral hemorrhage of rats. International immunopharmacology 2019; 75: 105771. doi: 10.1016/j.intimp.2019.105771 31352322

[24] Mitchell T, Rotaru D, Saba H, Smith RA, Murphy MP, MacMillan-Crow LA. The mitochondria-targeted antioxidant mitoquinone protects against cold storage injury of renal tubular cells and rat kidneys. The Journal of pharmacology and experimental therapeutics 2011; 336 (3): 682–92. doi: 10.1124/jpet.110.176743 21159749 PMC3382740

[25] Adler J, Parmryd I. Quantifying colocalization by correlation: the Pearson correlation coefficient is superior to the Mander’s overlap coefficient. Cytometry Part A: the journal of the International Society for Analytical Cytology 2010; 77 (8): 733–42. doi: 10.1002/cyto.a.20896 20653013

[26] Neag MA, Mitre AO, Catinean A, Mitre CI. An Overview on the Mechanisms of Neuroprotection and Neurotoxicity of Isoflurane and Sevoflurane in Experimental Studies. Brain research bulletin 2020; 165: 281–89. doi: 10.1016/j.brainresbull.2020.10.011 33080307

[27] Gao Y, Li J, Li J, Hu C, Zhang L, Yan J, et al. Tetrahydroxy stilbene glycoside alleviated inflammatory damage by mitophagy via AMPK related PINK1/Parkin signaling pathway. Biochemical pharmacology 2020; 177: 113997. doi: 10.1016/j.bcp.2020.113997 32353422

[28] Hu ZL, Sun T, Lu M, Ding JH, Du RH, Hu G. Kir6.1/K-ATP channel on astrocytes protects against dopaminergic neurodegeneration in the MPTP mouse model of Parkinson’s disease via promoting mitophagy. Brain, behavior, and immunity 2019; 81: 509–22. doi: 10.1016/j.bbi.2019.07.009 31288070

[29] Elliott EI, Sutterwala FS. Initiation and perpetuation of NLRP3 inflammasome activation and assembly. Immunological reviews 2015; 265 (1): 35–52. doi: 10.1111/imr.12286 25879282 PMC4400874

[30] Novak I. Mitophagy: a complex mechanism of mitochondrial removal. Antioxidants & redox signaling 2012; 17 (5): 794–802. doi: 10.1089/ars.2011.4407 22077334

[31] Kim MJ, Yoon JH, Ryu JH. Mitophagy: a balance regulator of NLRP3 inflammasome activation. BMB reports 2016; 49 (10): 529–35. doi: 10.5483/bmbrep.2016.49.10.115 27439607 PMC5227293

[32] Mohanty A, Tiwari-Pandey R, Pandey NR. Mitochondria: the indispensable players in innate immunity and guardians of the inflammatory response. Journal of cell communication and signaling 2019; 13 (3): 303–18. doi: 10.1007/s12079-019-00507-9 30719617 PMC6732146

[33] Jiang W, Liu F, Li H, Wang K, Cao X, Xu X, et al. TREM2 ameliorates anesthesia and surgery-induced cognitive impairment by regulating mitophagy and NLRP3 inflammasome in aged C57/BL6 mice. Neurotoxicology 2022; 90: 216–27. doi: 10.1016/j.neuro.2022.04.005 35447280

[34] Yu J, Nagasu H, Murakami T, Hoang H, Broderick L, Hoffman HM, et al. Inflammasome activation leads to Caspase-1-dependent mitochondrial damage and block of mitophagy. Proceedings of the National Academy of Sciences of the United States of America 2014; 111 (43): 15514–9. doi: 10.1073/pnas.1414859111 25313054 PMC4217429

[35] Jounai N, Kobiyama K, Shiina M, Ogata K, Ishii KJ, Takeshita F. NLRP4 negatively regulates autophagic processes through an association with beclin1. Journal of immunology 2011; 186 (3): 1646–55. doi: 10.4049/jimmunol.1001654 21209283

[36] Fu Q, Wu J, Zhou XY, Ji MH, Mao QH, Li Q, et al. NLRP3/Caspase-1 Pathway-Induced Pyroptosis Mediated Cognitive Deficits in a Mouse Model of Sepsis-Associated Encephalopathy. Inflammation 2019; 42 (1): 306–18. doi: 10.1007/s10753-018-0894-4 30276509 PMC6394578

[37] Zimering JH, Dong Y, Fang F, Huang L, Zhang Y, Xie Z. Anesthetic Sevoflurane Causes Rho-Dependent Filopodial Shortening in Mouse Neurons. PloS one 2016; 11 (7): e0159637. doi: 10.1371/journal.pone.0159637 27441369 PMC4956198

[38] Lu H, Liufu N, Dong Y, Xu G, Zhang Y, Shu L, et al. Sevoflurane Acts on Ubiquitination-Proteasome Pathway to Reduce Postsynaptic Density 95 Protein Levels in Young Mice. Anesthesiology 2017; 127 (6): 961–75. doi: 10.1097/ALN.0000000000001889 28968276 PMC5685882

[39] Magee JC, Grienberger C. Synaptic Plasticity Forms and Functions. Annual review of neuroscience 2020; 43: 95–117. doi: 10.1146/annurev-neuro-090919-022842 32075520

[40] Jin W, Lin D, Nguyen AH, Abdelrasoul GN, Chen J, Mar A, et al. Transfection of Difficult-to-Transfect Rat Primary Cortical Neurons with Magnetic Nanoparticles. Journal of biomedical nanotechnology 2018; 14 (9): 1654–64. doi: 10.1166/jbn.2018.2604 29958559

[41] Jessen KR. Glial cells. The international journal of biochemistry & cell biology 2004; 36 (10): 1861–7. doi: 10.1016/j.biocel.2004.02.023 15203098

[42] Jia R, Bonifacino JS. Negative regulation of autophagy by UBA6-BIRC6-mediated ubiquitination of LC3. eLife 2019; 8. doi: 10.7554/eLife.50034 31692446 PMC6863627

[43] Li S, Sun Y, Song M, Song Y, Fang Y, Zhang Q, et al. NLRP3/caspase-1/GSDMD-mediated pyroptosis exerts a crucial role in astrocyte pathological injury in mouse model of depression. JCI insight 2021; 6 (23). doi: 10.1172/jci.insight.146852 34877938 PMC8675200

[44] Liu X, Zhang Z, Ruan J, Pan Y, Magupalli VG, Wu H, et al. Inflammasome-activated gasdermin D causes pyroptosis by forming membrane pores. Nature 2016; 535 (7610): 153–8. doi: 10.1038/nature18629 27383986 PMC5539988

[45] Sborgi L, Ruhl S, Mulvihill E, Pipercevic J, Heilig R, Stahlberg H, et al. GSDMD membrane pore formation constitutes the mechanism of pyroptotic cell death. The EMBO journal 2016; 35 (16): 1766–78. doi: 10.15252/embj.201694696 27418190 PMC5010048

