# Supplementary figures and images for: Ac-YVAD-cmk ameliorated sevoflurane-induced cognitive dysfunction and revised mitophagy impairment
Source: PLoS One. 2023 Jan 25;18(1):e0280914. doi: 10.1371/journal.pone.0280914 (PMC9876368; doi:10.1371/journal.pone.0280914)

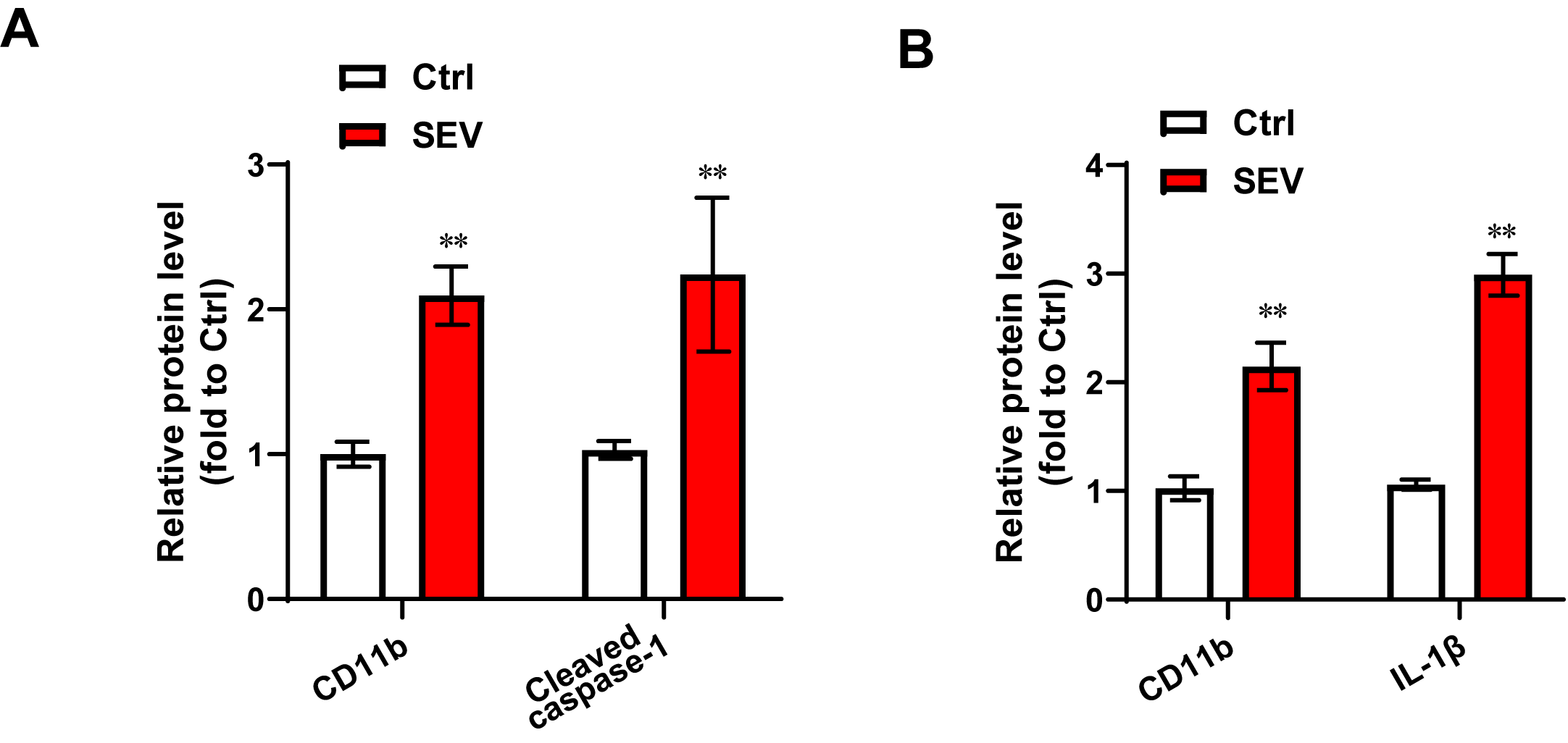

Supplement: S1 Fig — (A) The results of semi-quantitative analysis of Cleaved caspase-1 and CD11b are shown. (B) The results of semi-quantitative analysis of IL-1β and CD11b are shown. The data are expressed as mean ± SD. * P<0.05, **P<0.01, Ctrl vs SEV. The experiment was repeated for three times. (TIF) [file pone.0280914.s001.tif]

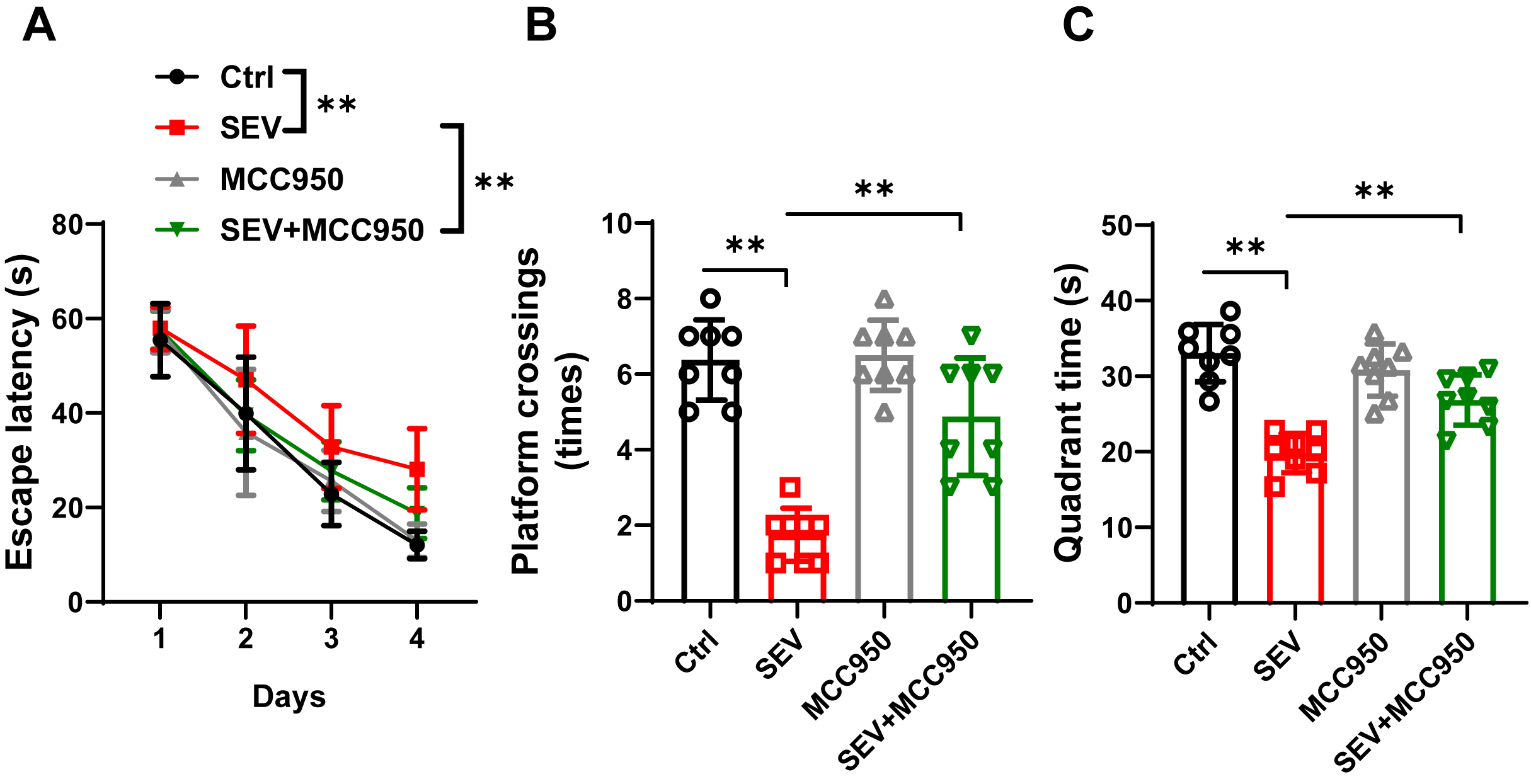

Supplement: S2 Fig — Eighteen-month-old mice were subjected to 2% sevoflurane for 5 h. aged mice were injected intraperitoneally (i.p.) with 50 mg/kg MCC950 or vehicle control (DMSO/PBS) 1 h h before sevoflurane treatment. The Morris Water Maze was used to test the learning ability. The parameters escape latency (A) number of platform crossings (B) and quadrant time (C) were measured. n = 8. The data are expressed as mean ± SD. * P<0.05, **P<0.01. (TIF) [file pone.0280914.s002.tif]

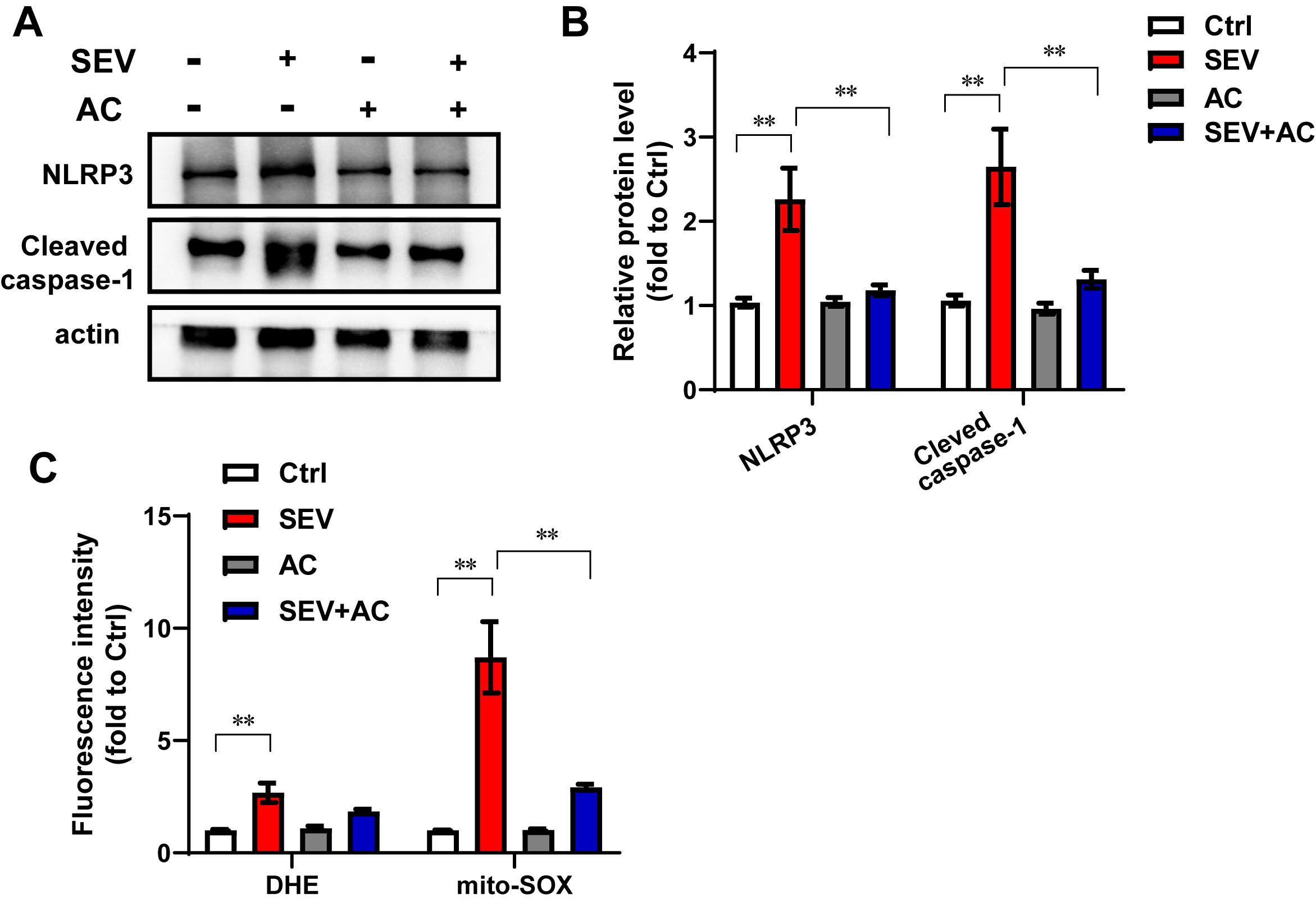

Supplement: S3 Fig — (A) Comparison of NLRP3 and Cleaved caspas-1 expression in each group. β-actin was used as an endogenous control. (B) The semi-quantitative analysis for the blotting. n = 6; (C) The intracellular ROS and mitochondrial ROS levels were measured. n = 9. The data are expressed as mean ± SD. * P<0.05, **P<0.01. (TIF) [file pone.0280914.s003.tif]

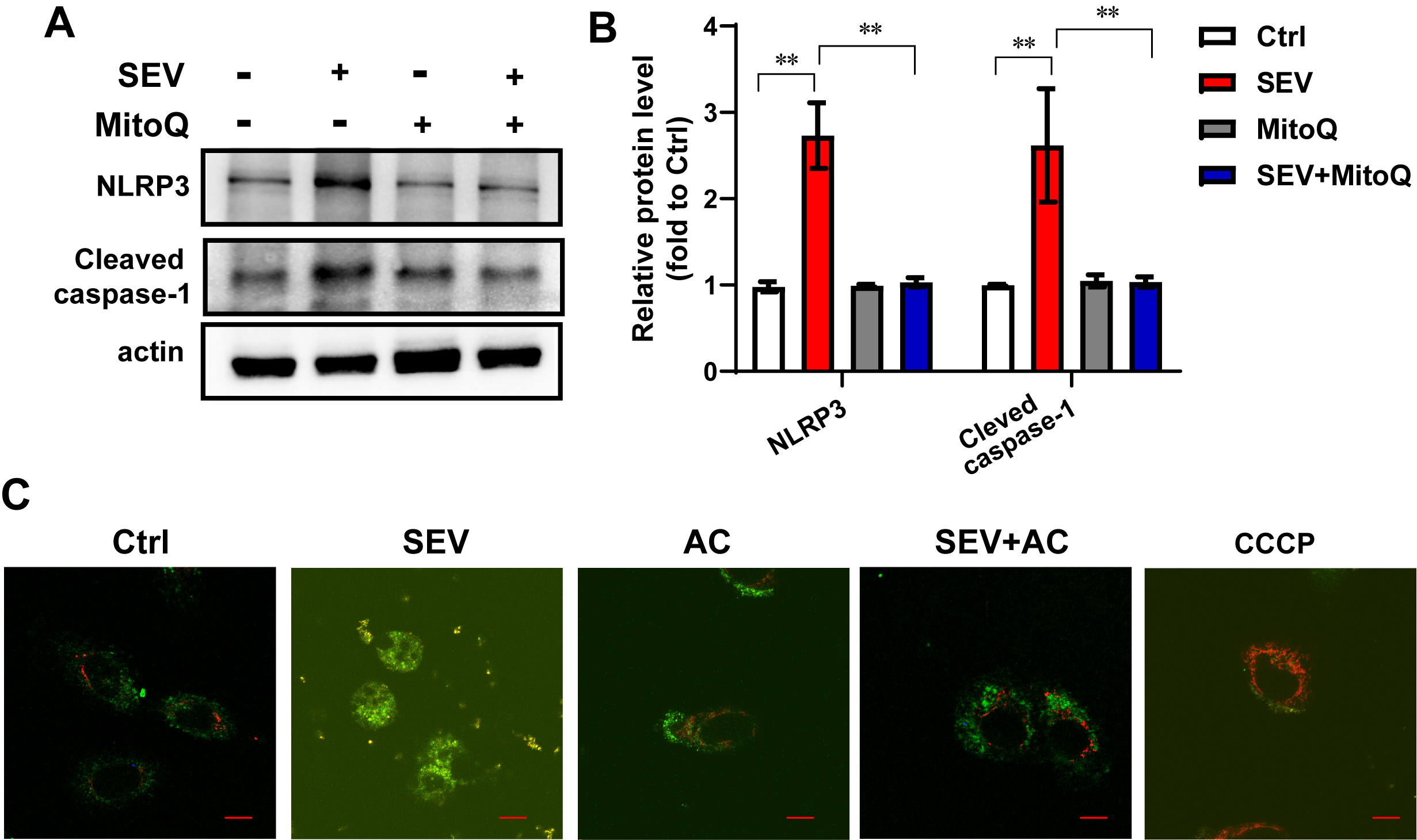

Supplement: S4 Fig — (A) Comparison of NLRP3 and Cleaved caspas-1 expression in each group. β-actin was used as an endogenous control. (B) The semi-quantitative analysis for the blotting. n = 6; (C) The more images of the mito-Keima in Fig 5B were shown. The image of the mito-Keima after CCCP treatment was also shown as positive control. The data are expressed as mean ± SD. * P<0.05, **P<0.01. (TIF) [file pone.0280914.s004.tif]

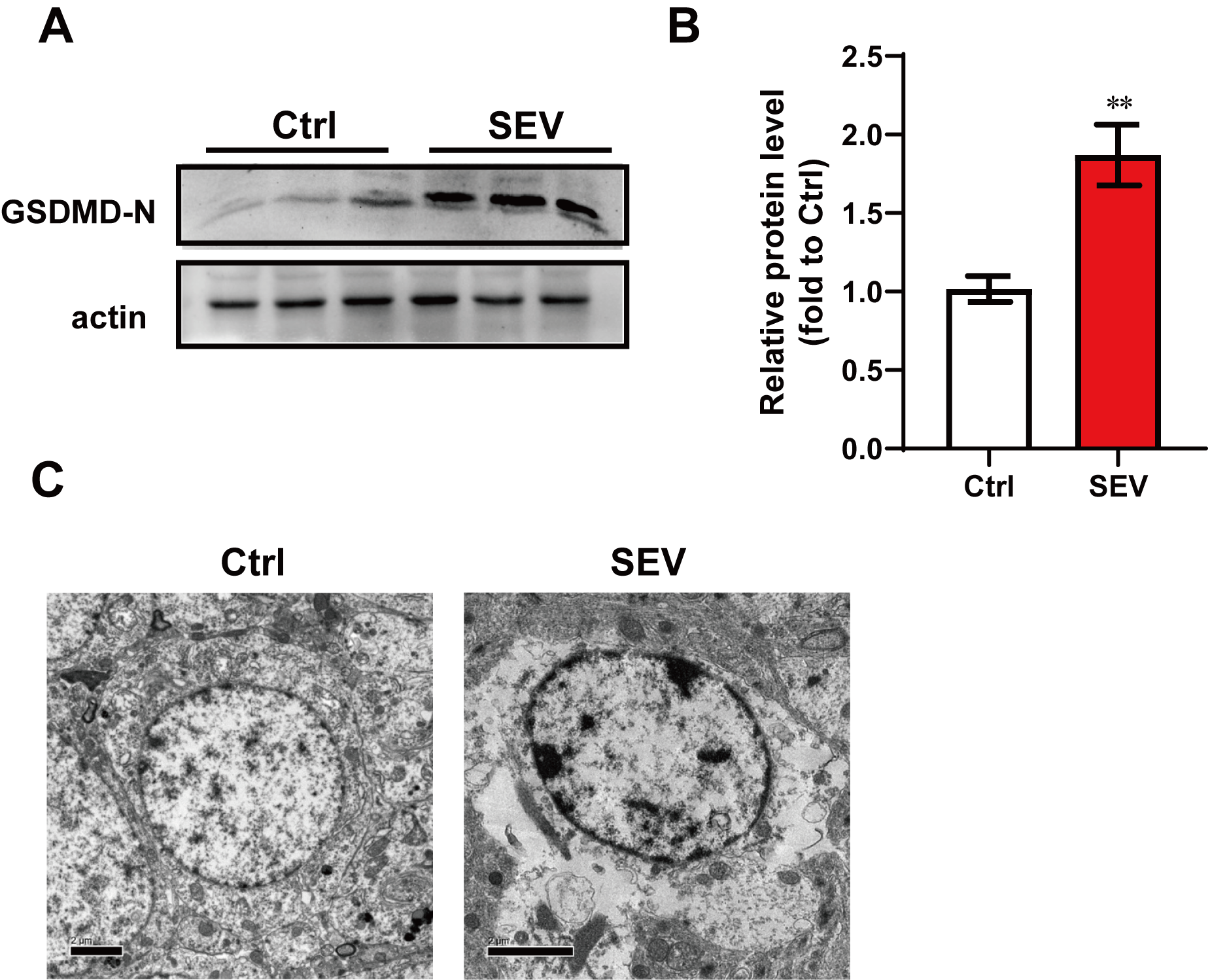

Supplement: S5 Fig — The hippocampus of all the aged mice were harvested. (A) Comparison of Cleaved GSDMD expression in the hippocampus of aged mice in each group. β-actin was used as an endogenous control; (B) The semi-quantitative analysis for the blotting; n = 6; (C) the ultrastructural damages of cells in hippocampus. n = 3; The data are expressed as mean ± SD. **P<0.01. (TIF) [file pone.0280914.s005.tif]

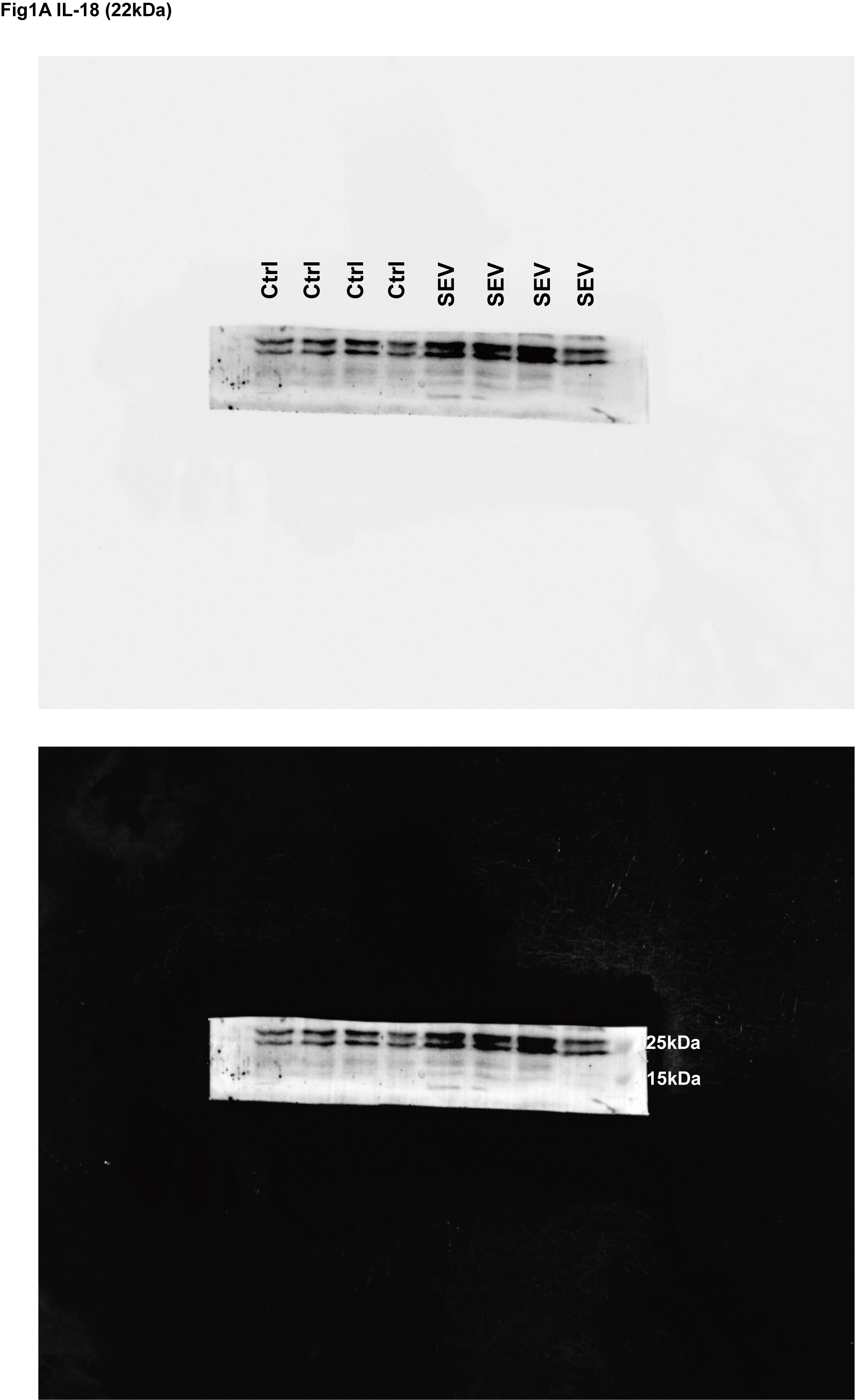

Supplement: S1 Raw images — (ZIP) [file pone.0280914.s006.zip › fig1A_raw_images/fig1A-IL-18.tif]

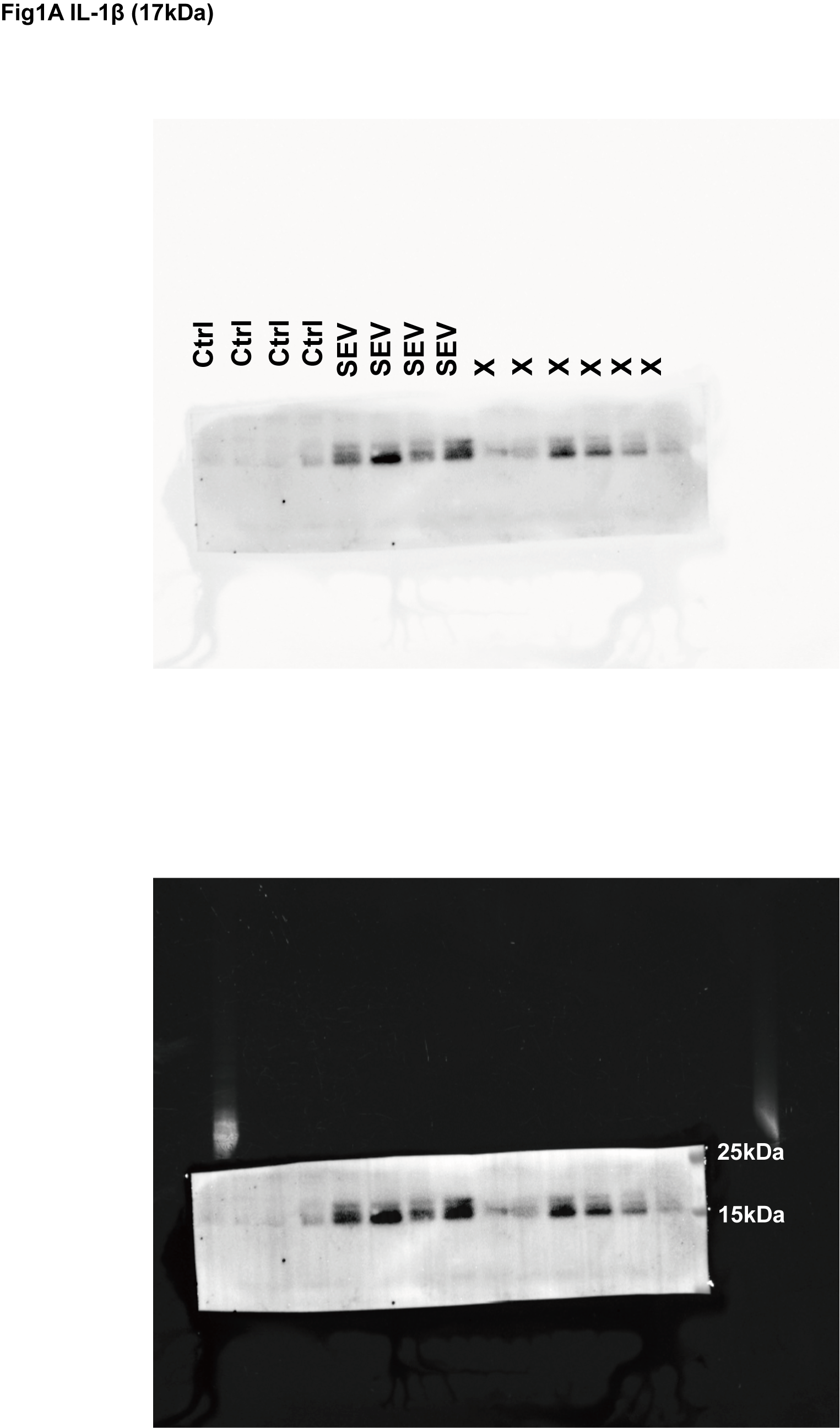

Supplement: S1 Raw images — (ZIP) [file pone.0280914.s006.zip › fig1A_raw_images/fig1A-IL-1b.tif]

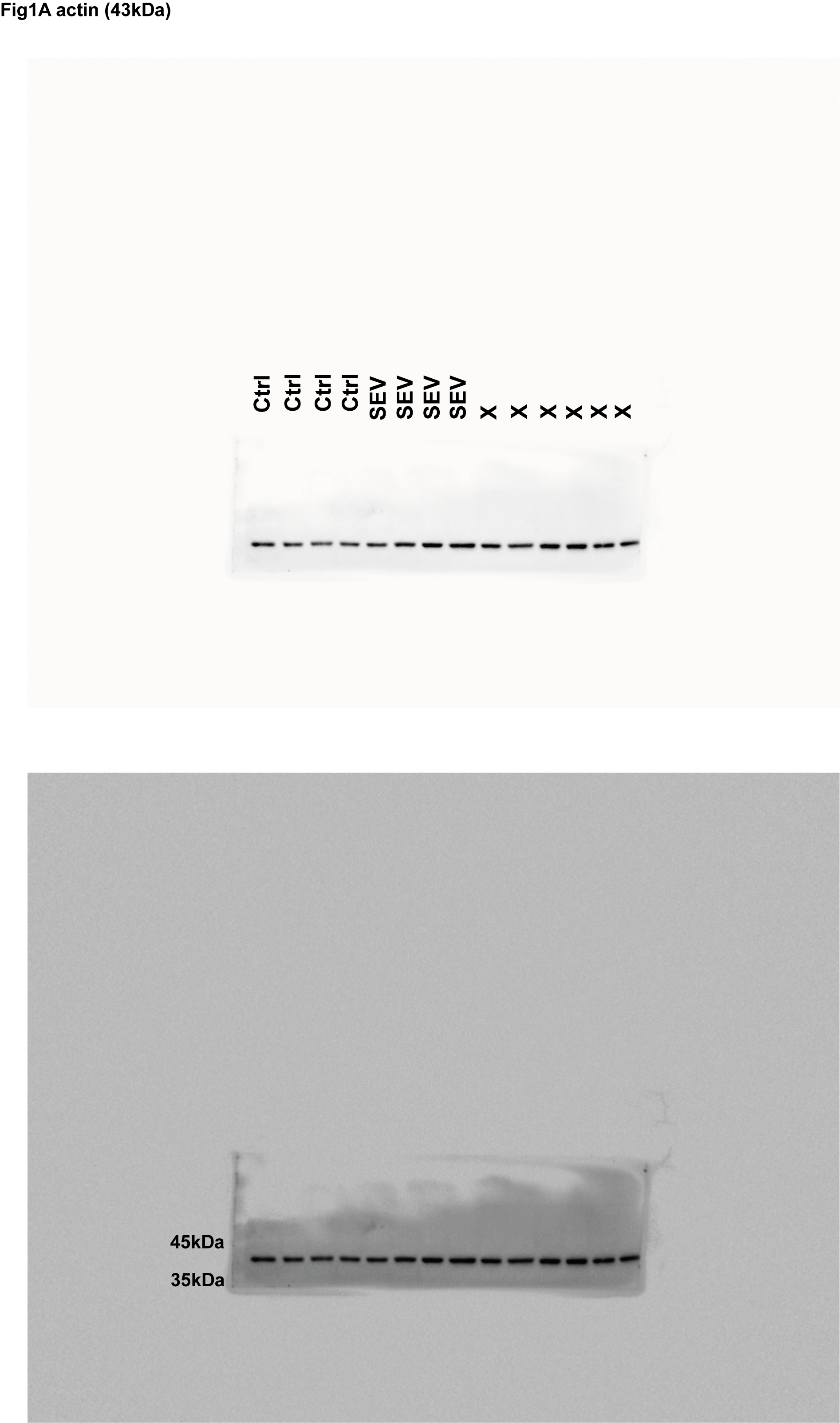

Supplement: S1 Raw images — (ZIP) [file pone.0280914.s006.zip › fig1A_raw_images/fig1A-actin.tif]

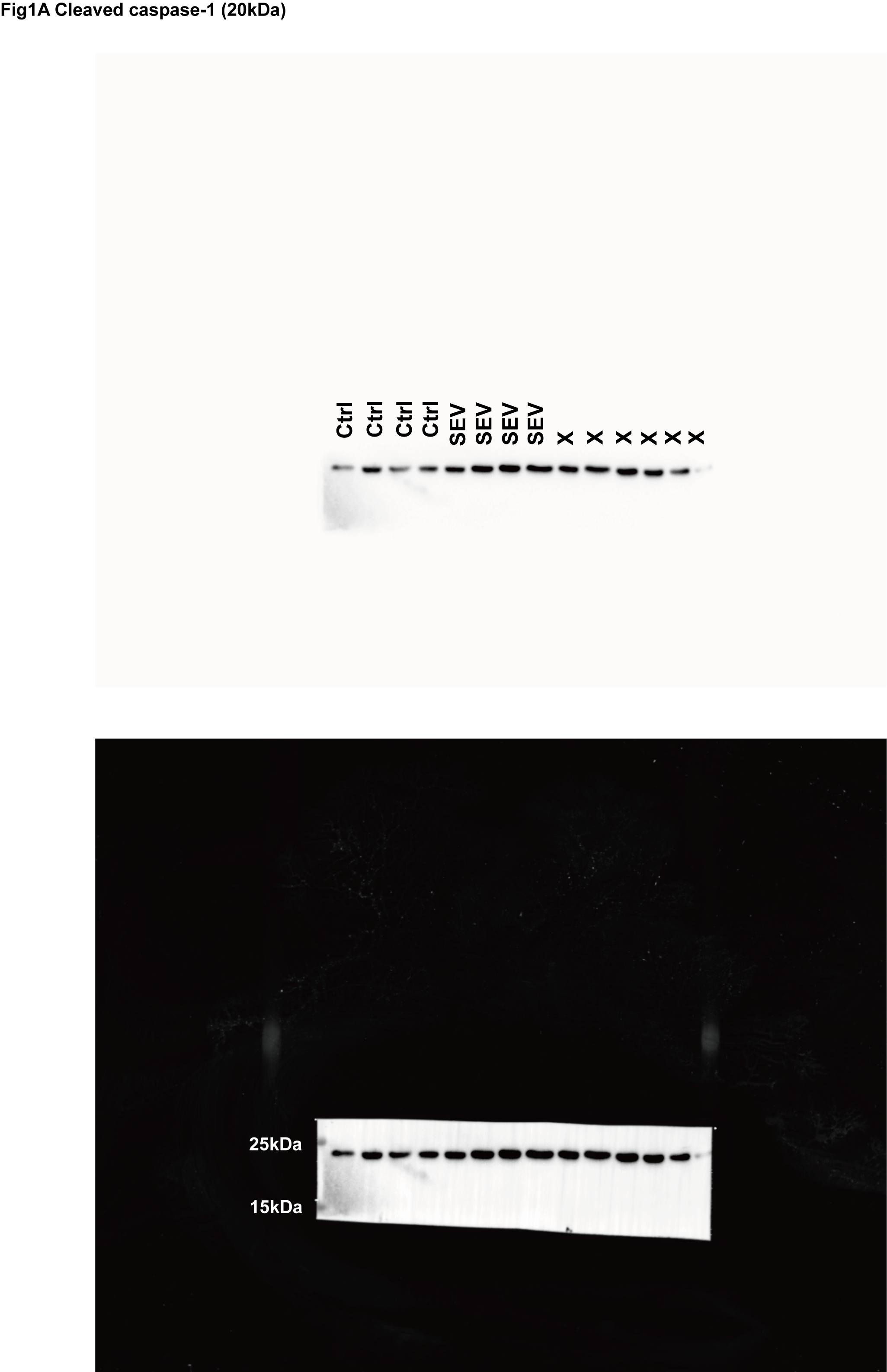

Supplement: S1 Raw images — (ZIP) [file pone.0280914.s006.zip › fig1A_raw_images/fig1A-caspase-1.tif]

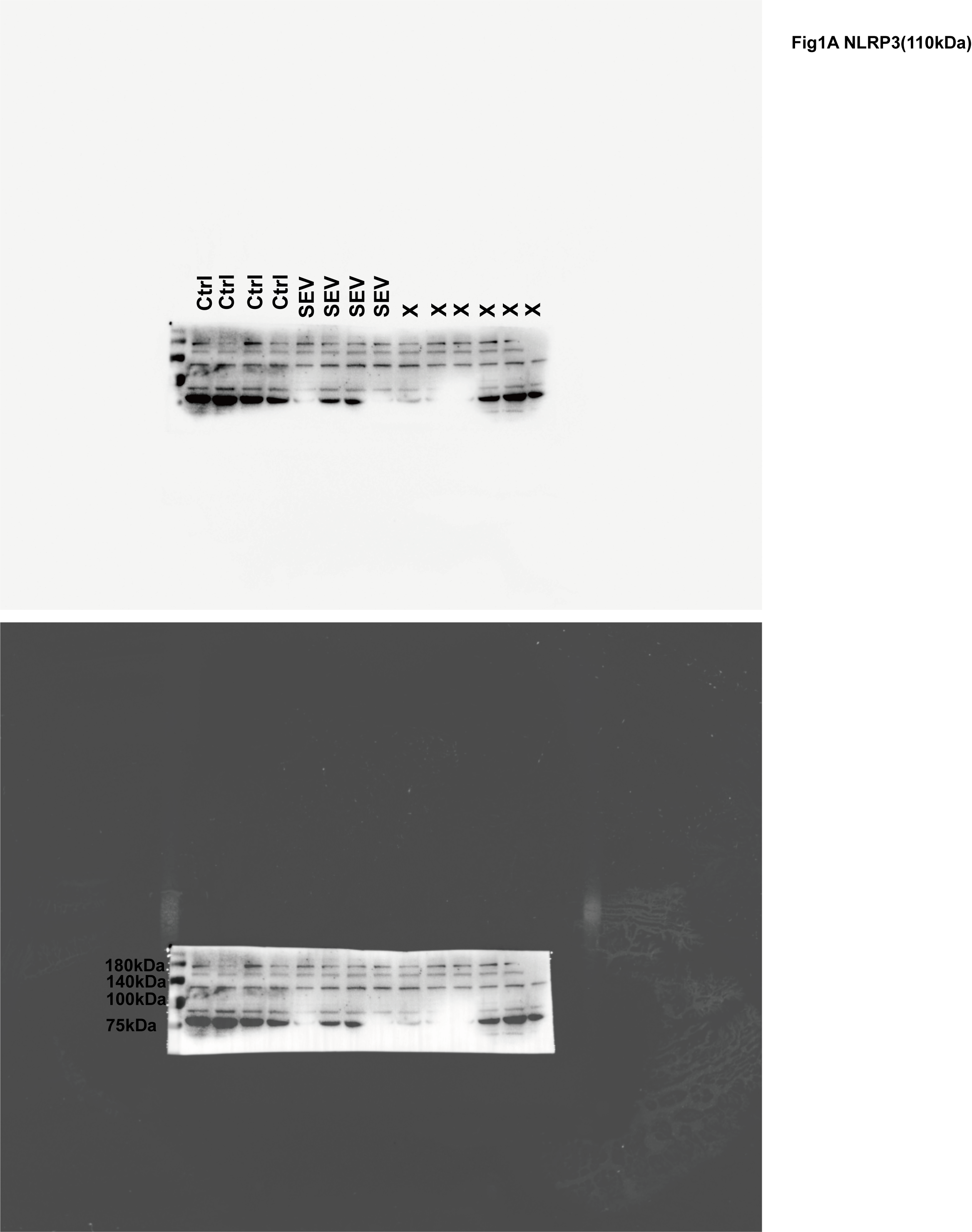

Supplement: S1 Raw images — (ZIP) [file pone.0280914.s006.zip › fig1A_raw_images/fig1A-nlrp3.tif]

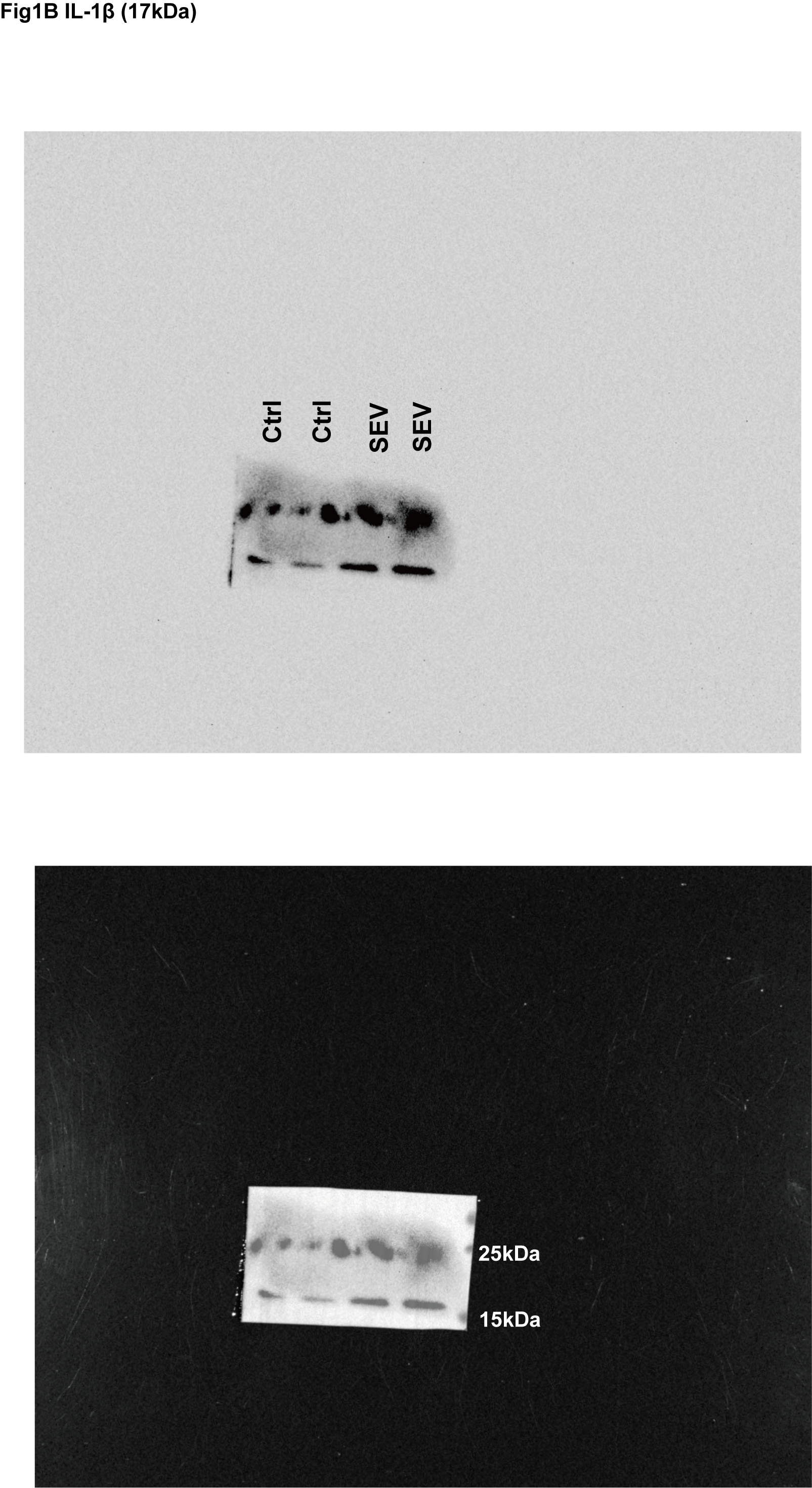

Supplement: S2 Raw images — (ZIP) [file pone.0280914.s007.zip › fig1B_raw_images/fig1B-IL-1b.tif]

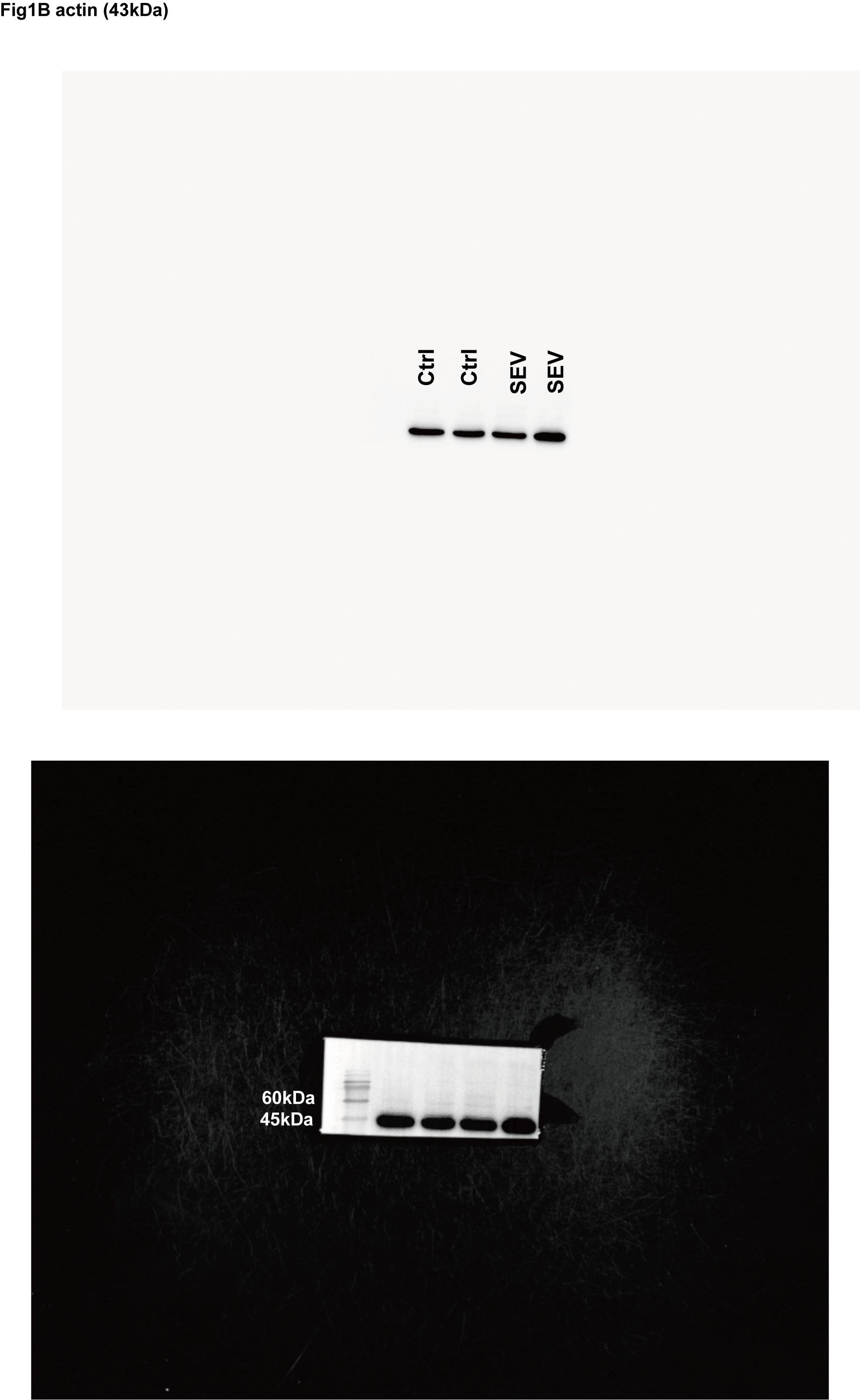

Supplement: S2 Raw images — (ZIP) [file pone.0280914.s007.zip › fig1B_raw_images/fig1B-actin.tif]

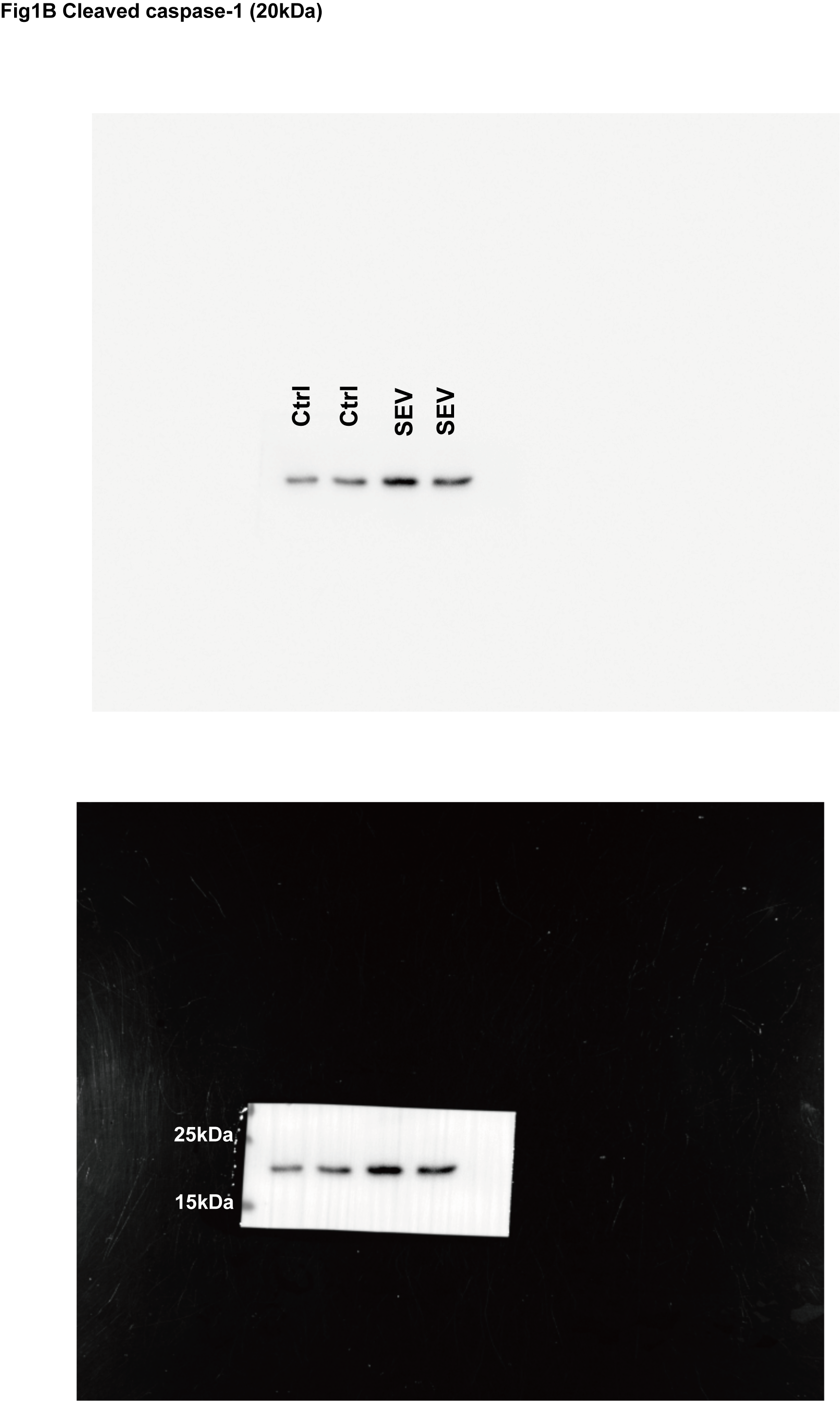

Supplement: S2 Raw images — (ZIP) [file pone.0280914.s007.zip › fig1B_raw_images/fig1B-caspase-1.tif]

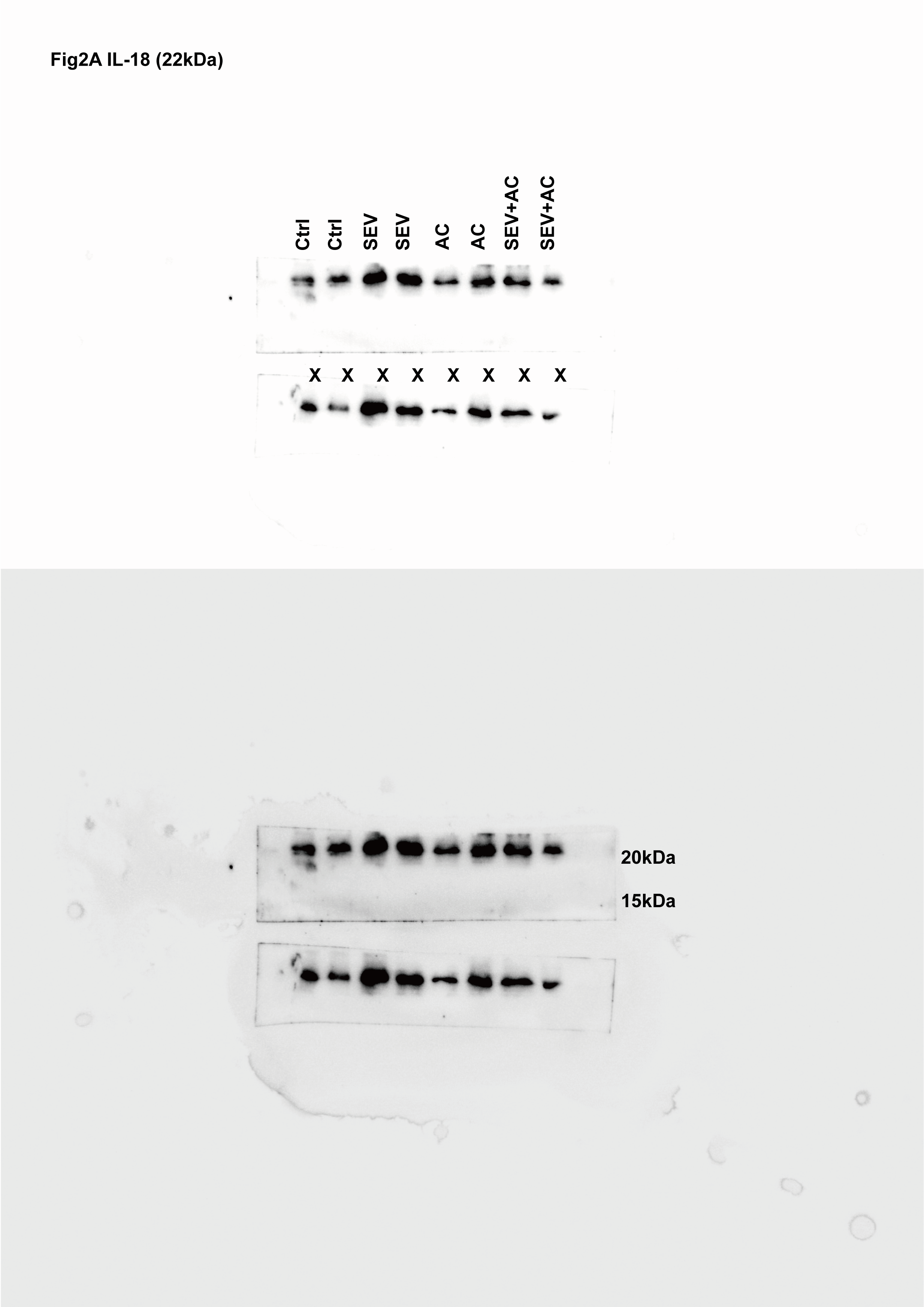

Supplement: S3 Raw images — (ZIP) [file pone.0280914.s008.zip › fig2A_raw_images/fig2-IL-18.tif]

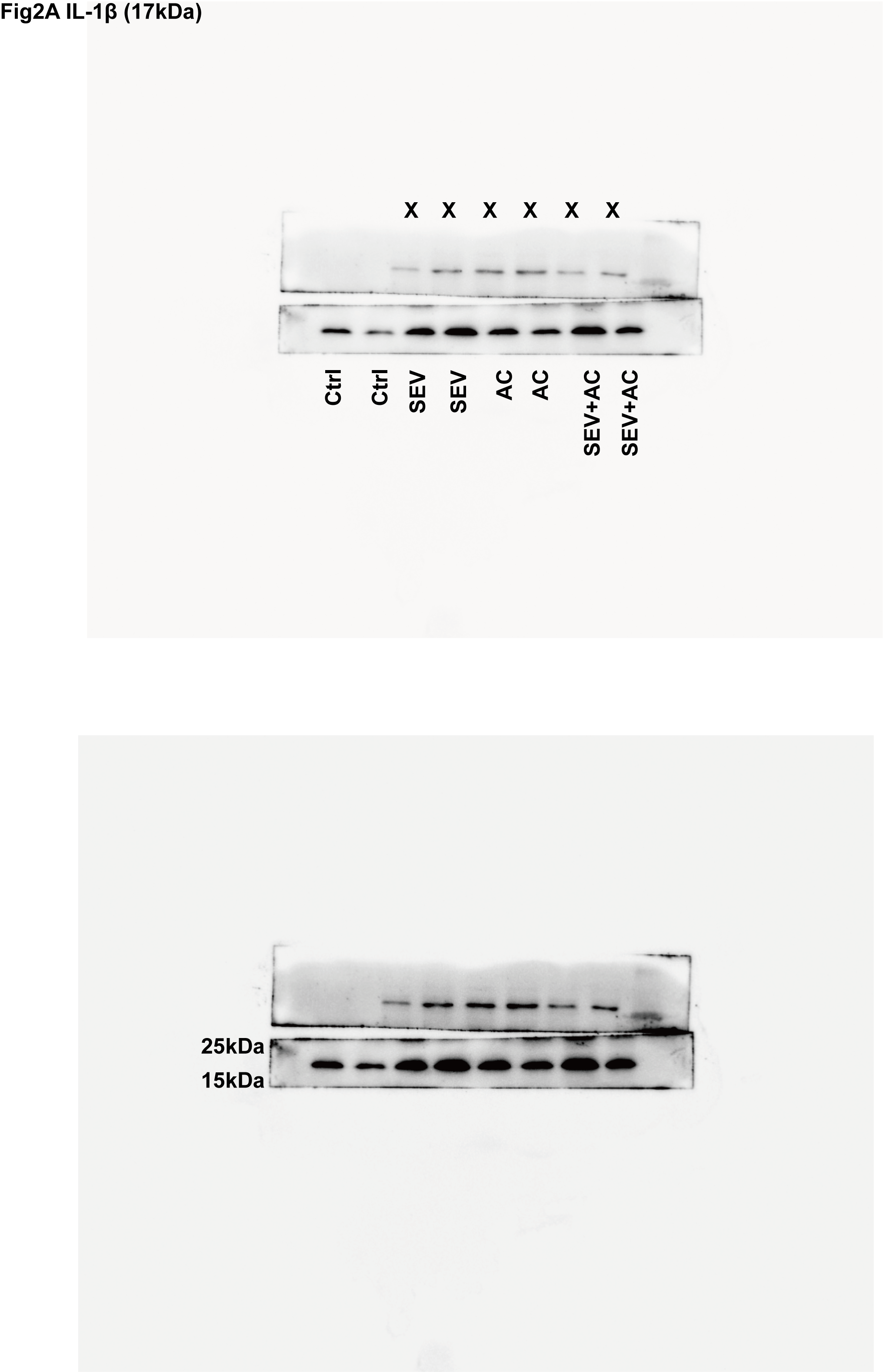

Supplement: S3 Raw images — (ZIP) [file pone.0280914.s008.zip › fig2A_raw_images/fig2-IL-1b.tif]

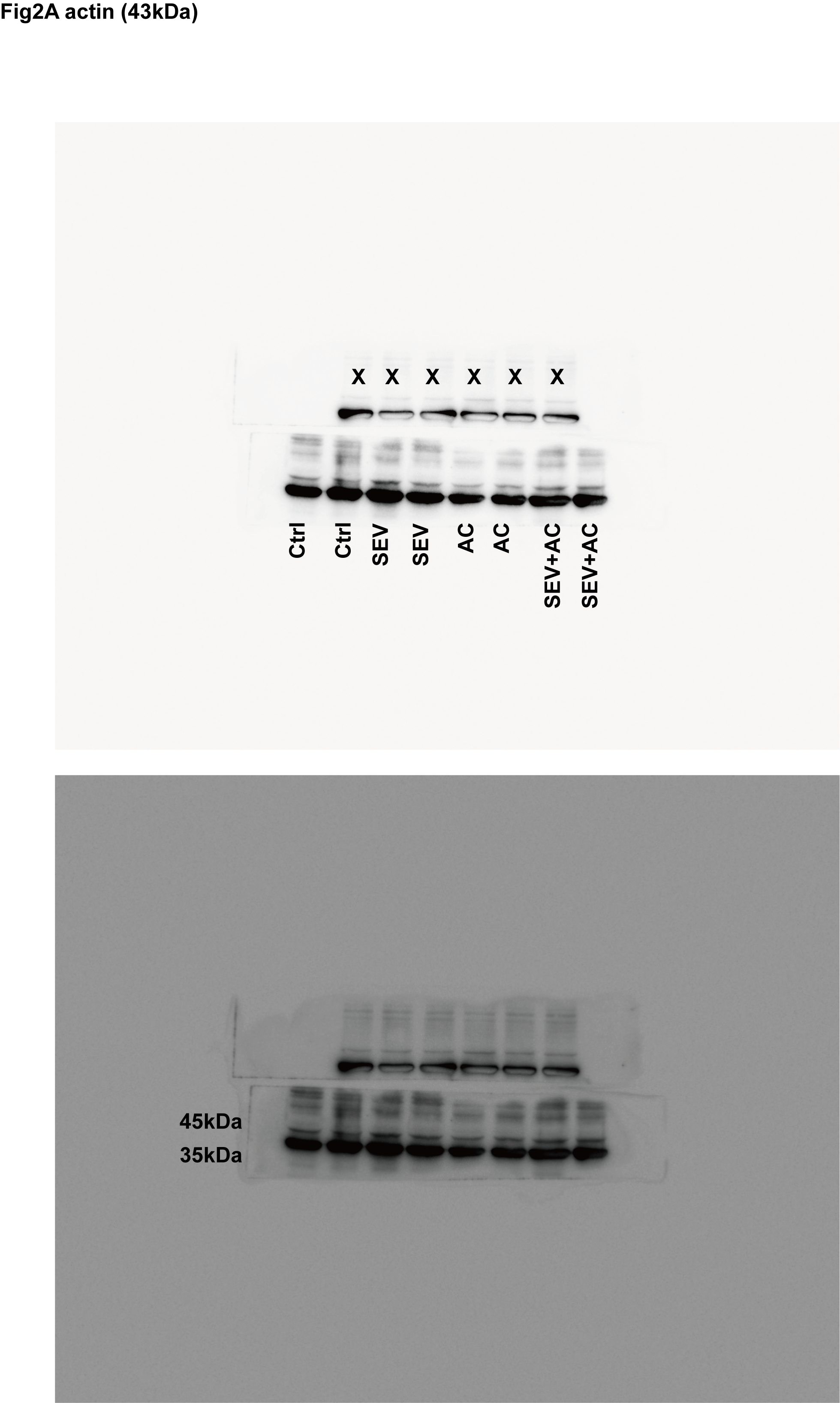

Supplement: S3 Raw images — (ZIP) [file pone.0280914.s008.zip › fig2A_raw_images/fig2-actin.tif]

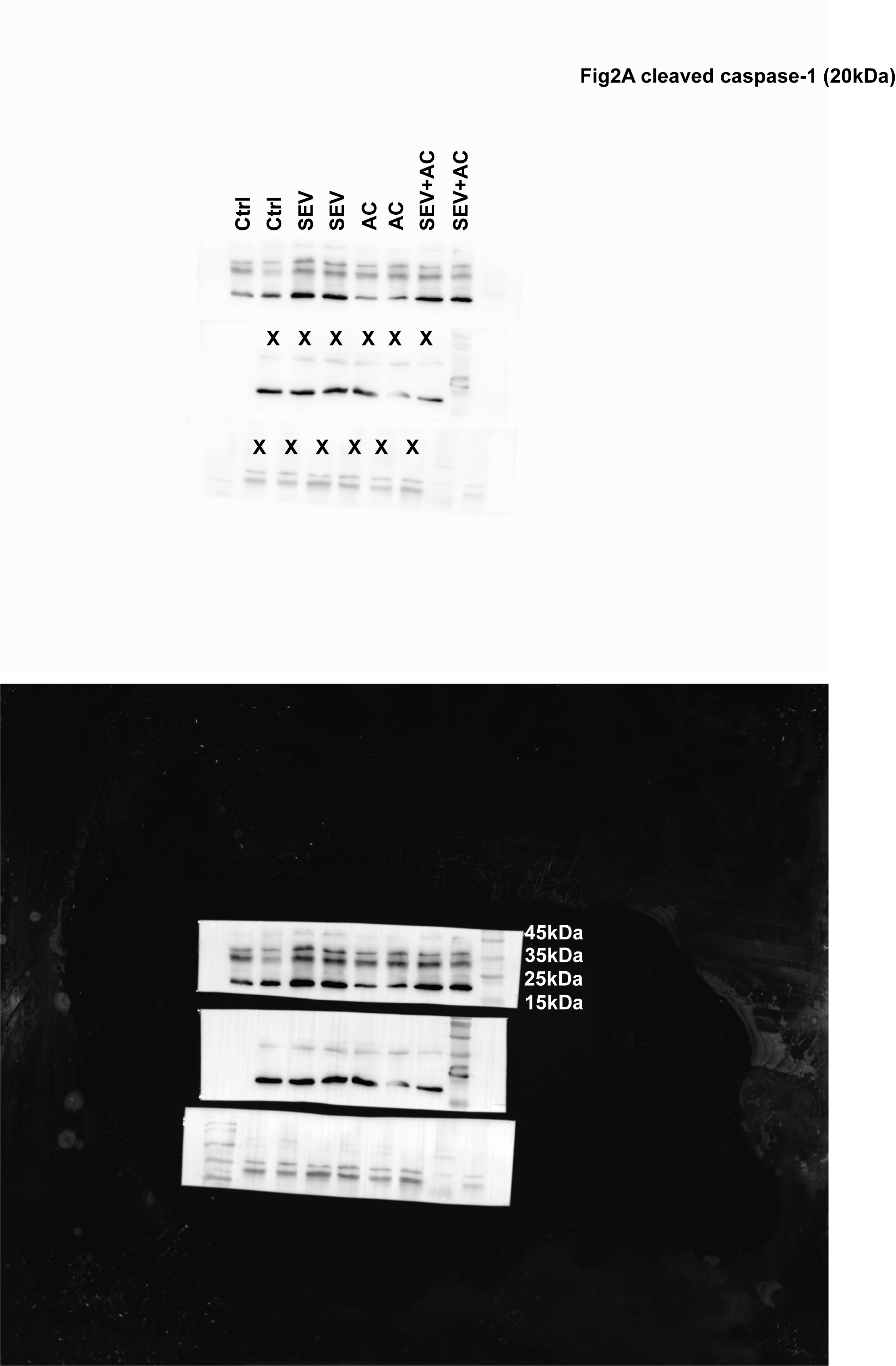

Supplement: S3 Raw images — (ZIP) [file pone.0280914.s008.zip › fig2A_raw_images/fig2-caspas-1.tif]

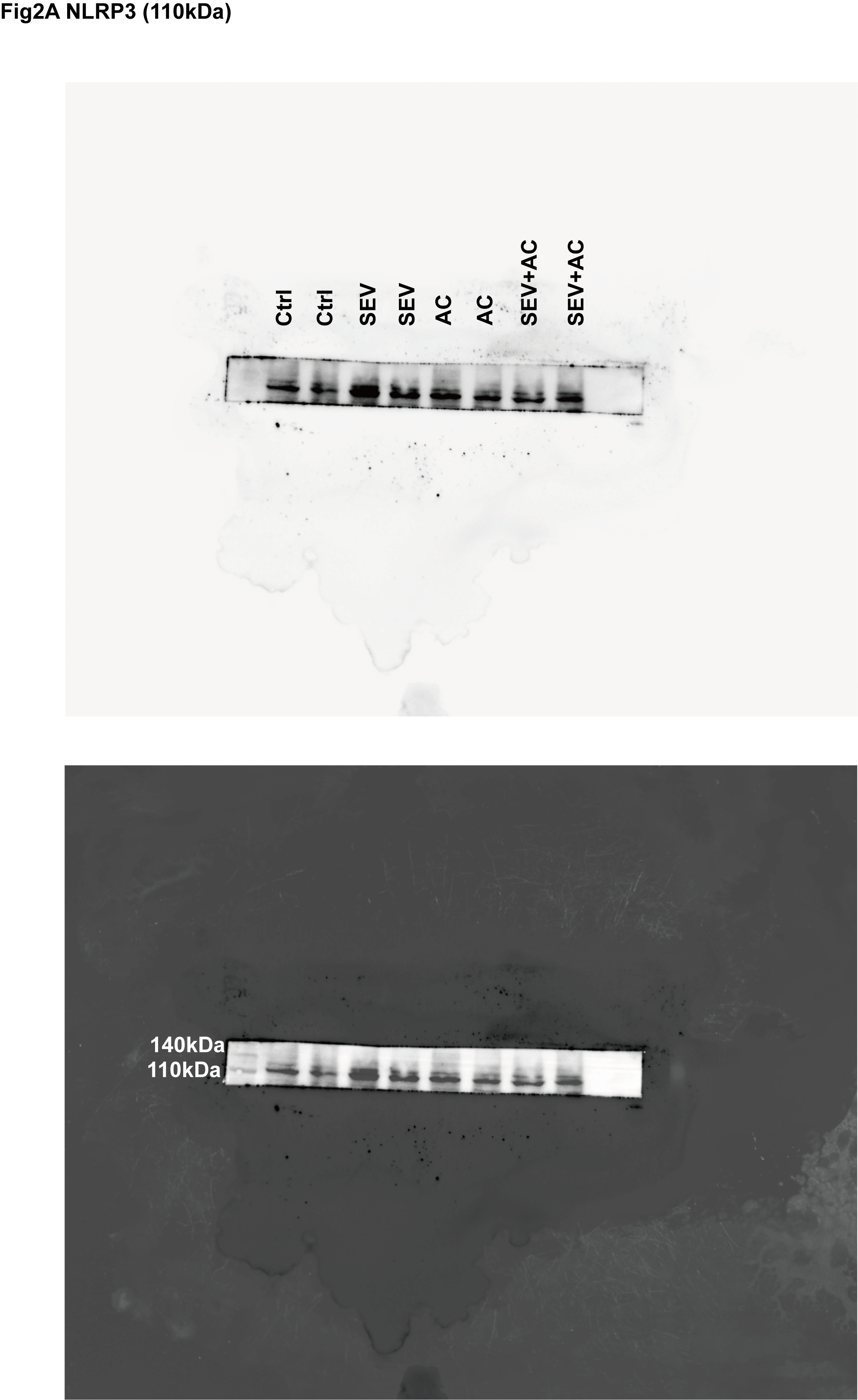

Supplement: S3 Raw images — (ZIP) [file pone.0280914.s008.zip › fig2A_raw_images/fig2-nlrp3.tif]

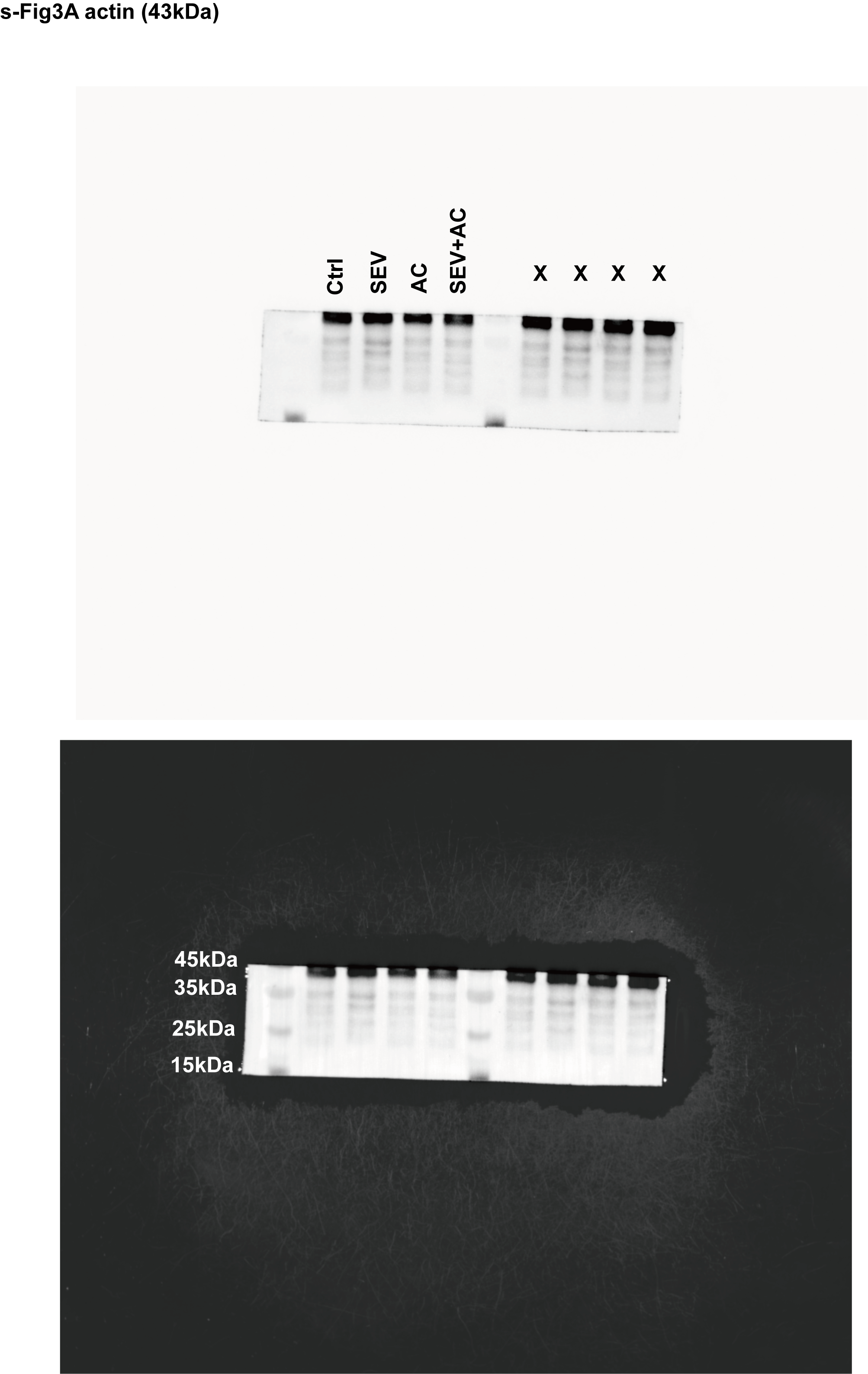

Supplement: S4 Raw images — (ZIP) [file pone.0280914.s009.zip › s-fig3A_raw_images/s-fig3-actin.tif]

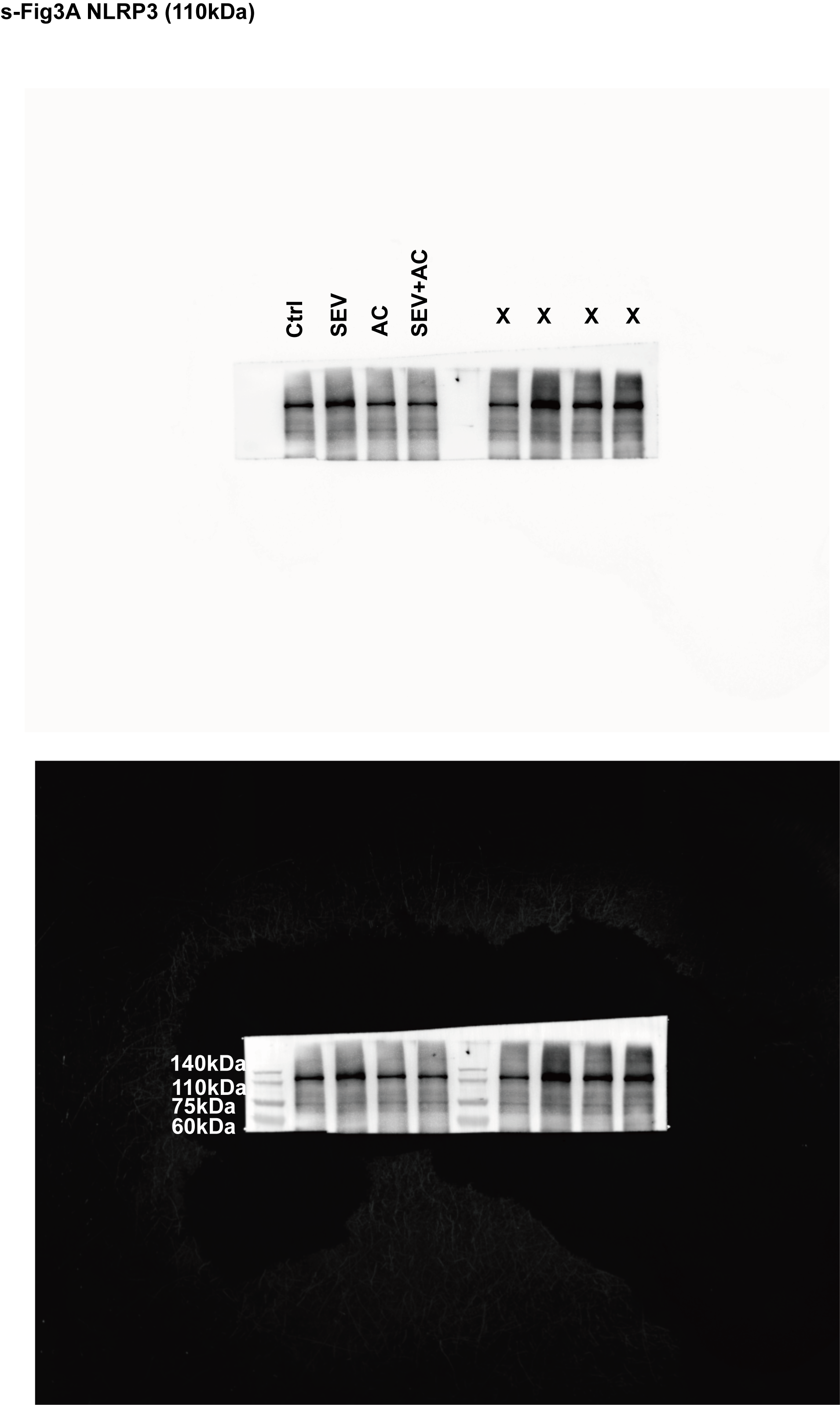

Supplement: S4 Raw images — (ZIP) [file pone.0280914.s009.zip › s-fig3A_raw_images/s-fig3A-NLRP3.tif]

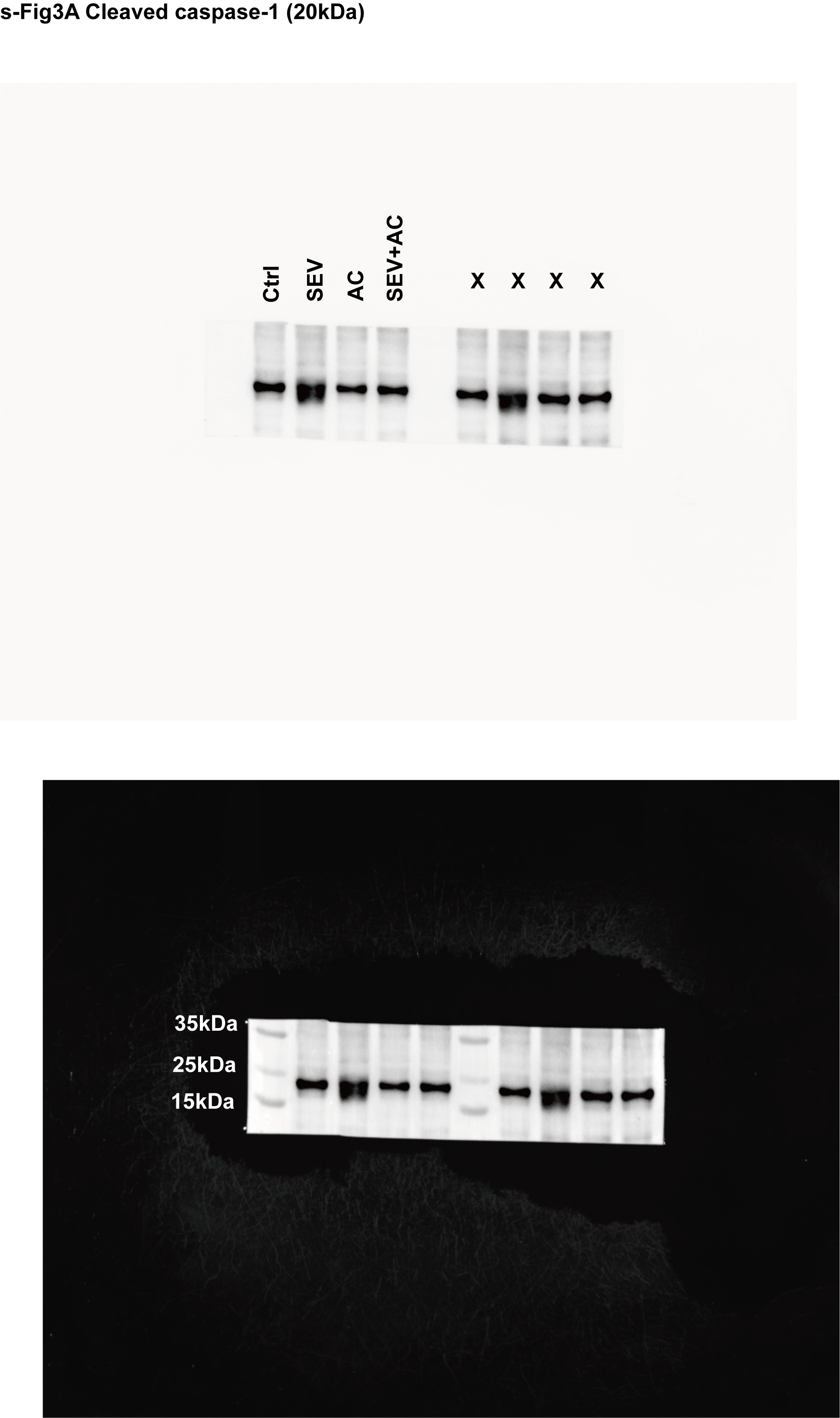

Supplement: S4 Raw images — (ZIP) [file pone.0280914.s009.zip › s-fig3A_raw_images/s-fig3A-caspase-1.tif]

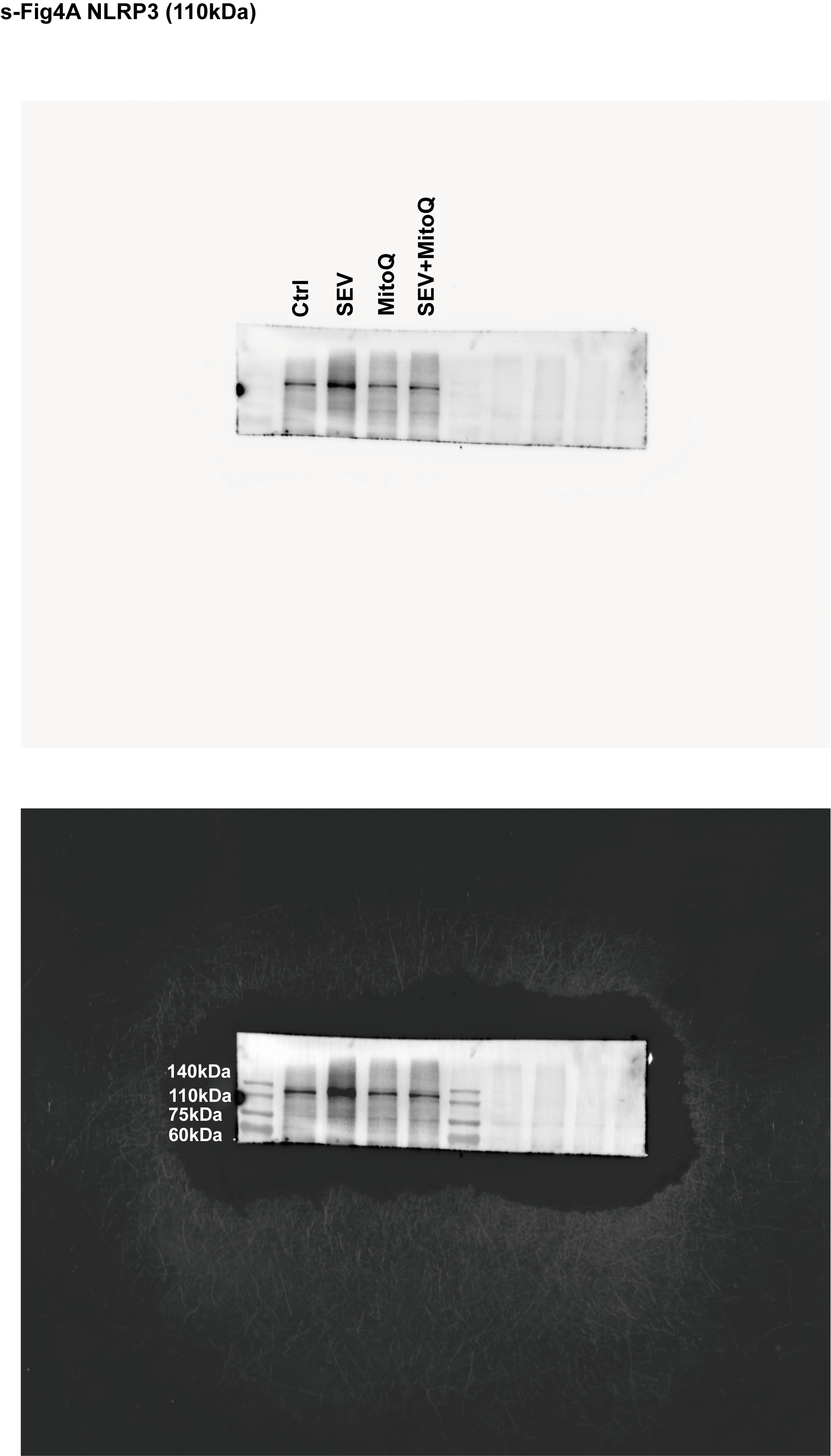

Supplement: S5 Raw images — (ZIP) [file pone.0280914.s010.zip › s-fig4A_raw_images/s-fig4A-NLRP3.tif]

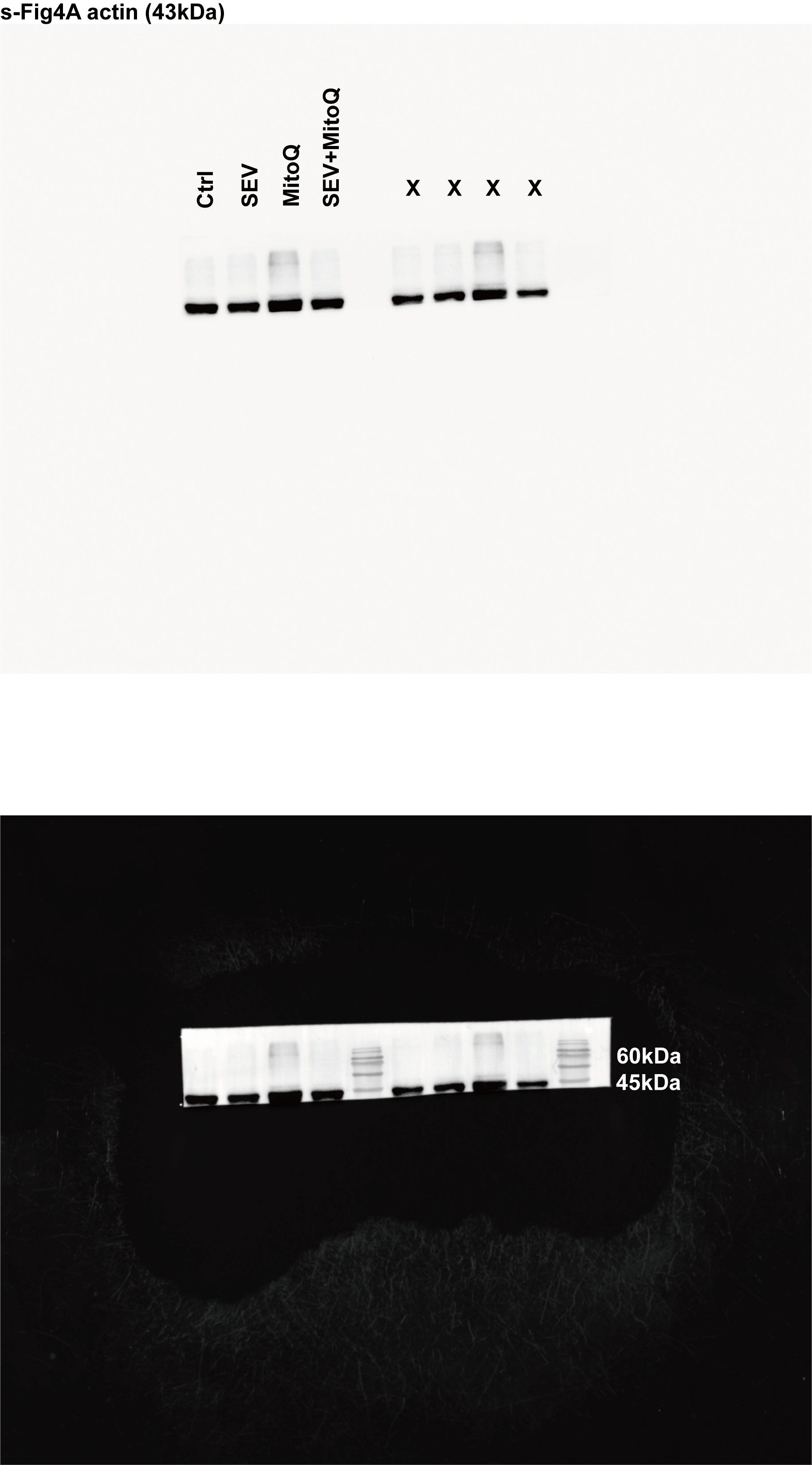

Supplement: S5 Raw images — (ZIP) [file pone.0280914.s010.zip › s-fig4A_raw_images/s-fig4A-actin.tif]

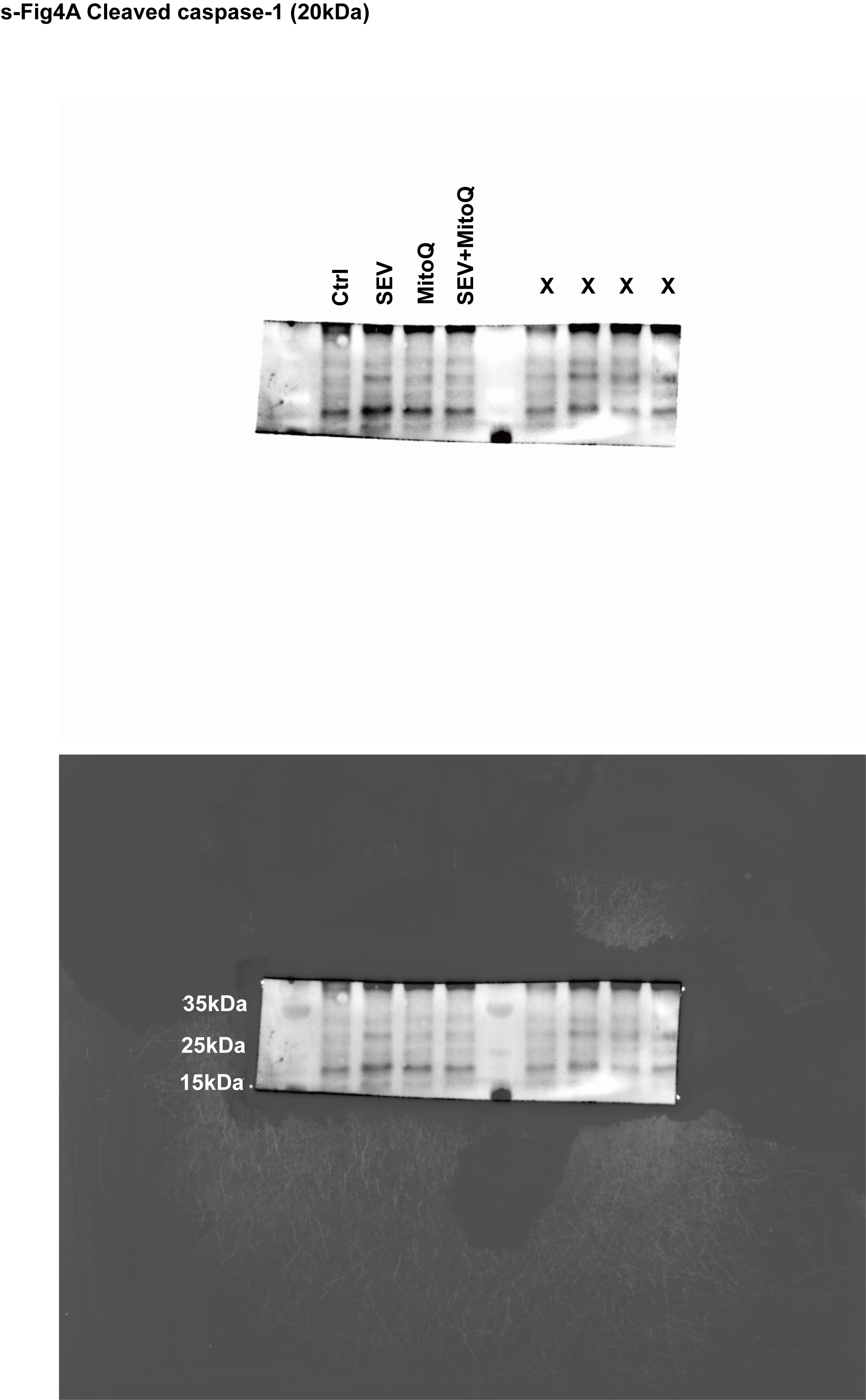

Supplement: S5 Raw images — (ZIP) [file pone.0280914.s010.zip › s-fig4A_raw_images/s-fig4A-caspase-1.tif]

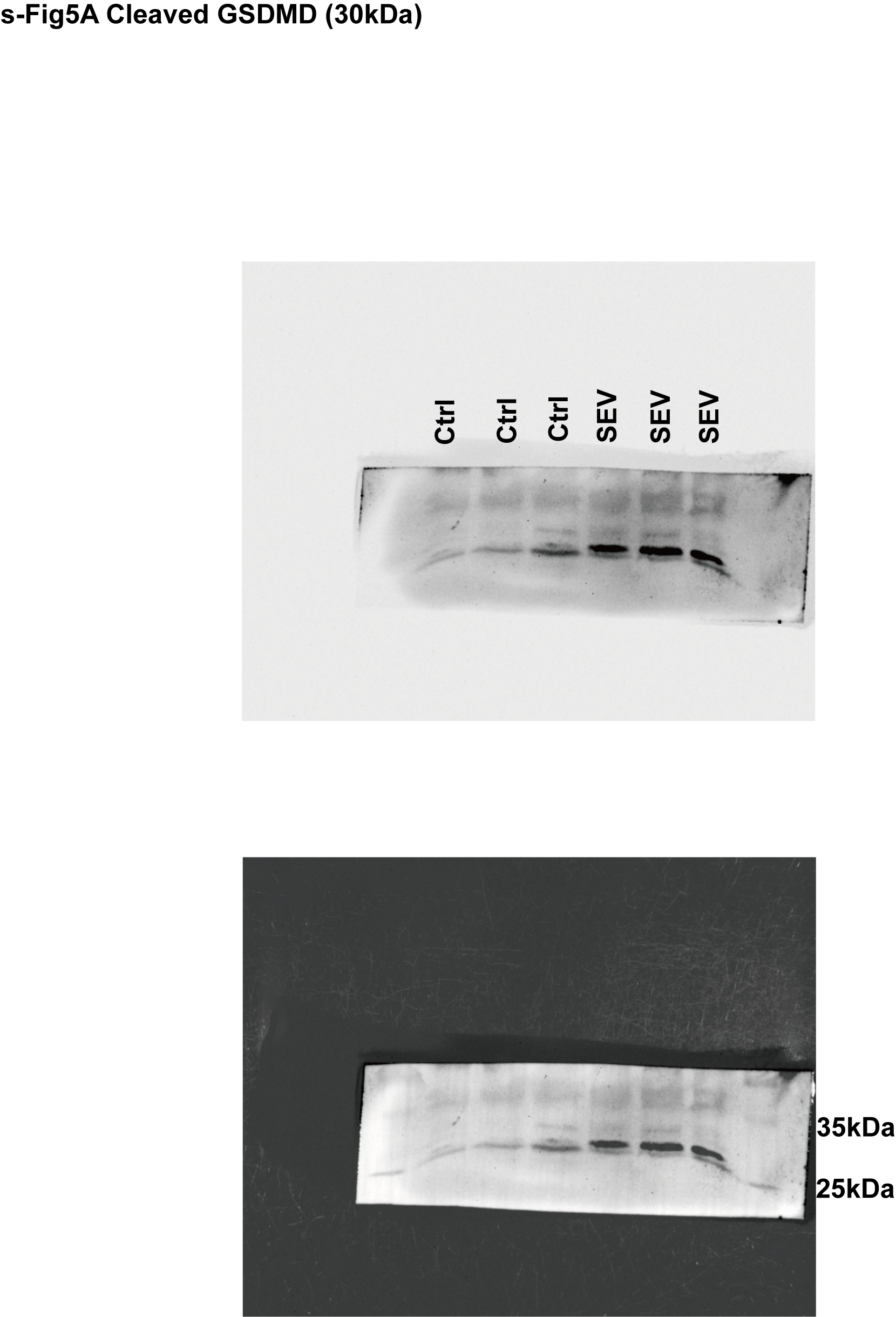

Supplement: S6 Raw images — (ZIP) [file pone.0280914.s011.zip › s-fig5A_raw_images/s-fig5-GSDMD.tif]

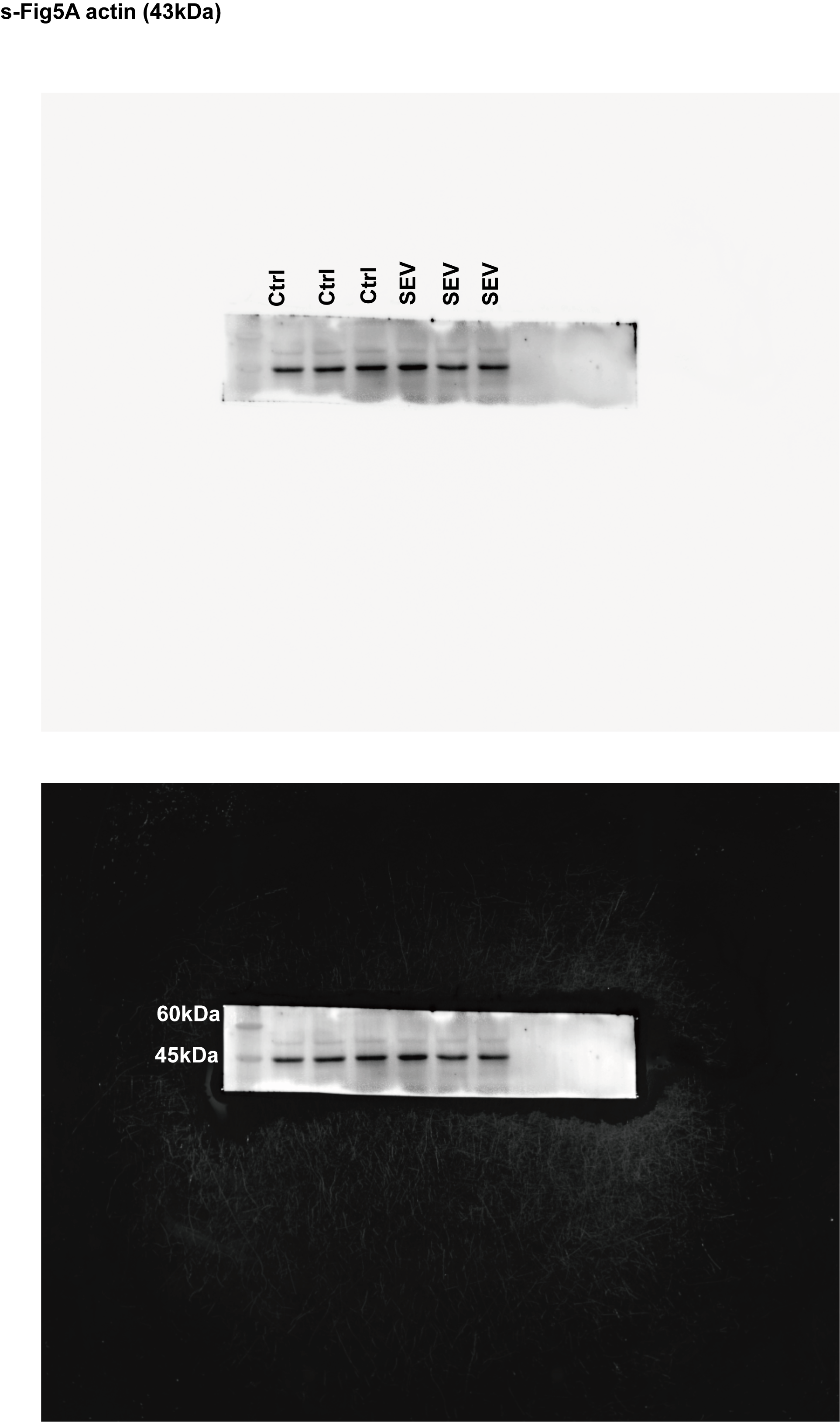

Supplement: S6 Raw images — (ZIP) [file pone.0280914.s011.zip › s-fig5A_raw_images/s-fig5-actin.tif]

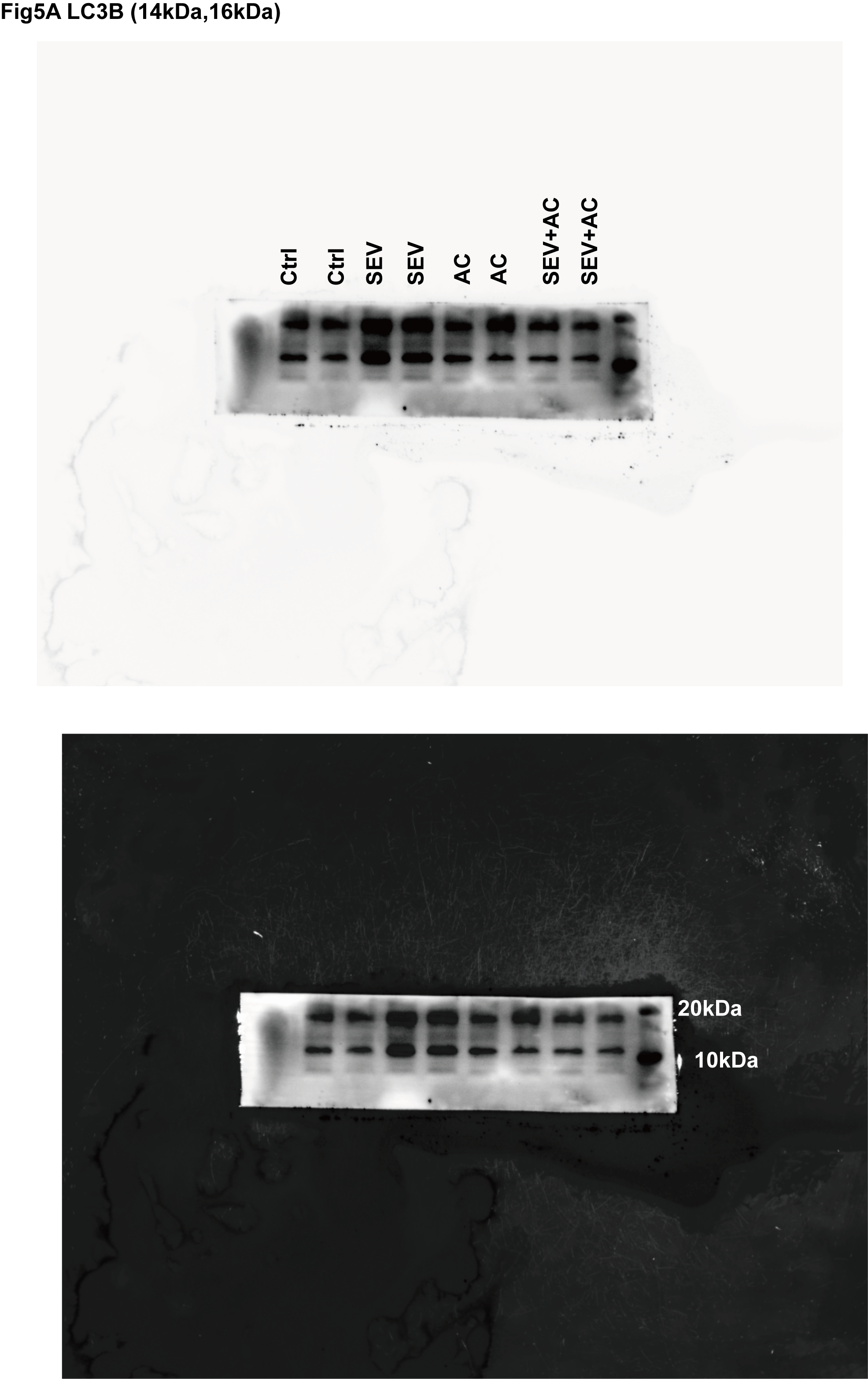

Supplement: S7 Raw images — (ZIP) [file pone.0280914.s012.zip › fig5A_raw_images/fig5A-LC3B.tif]

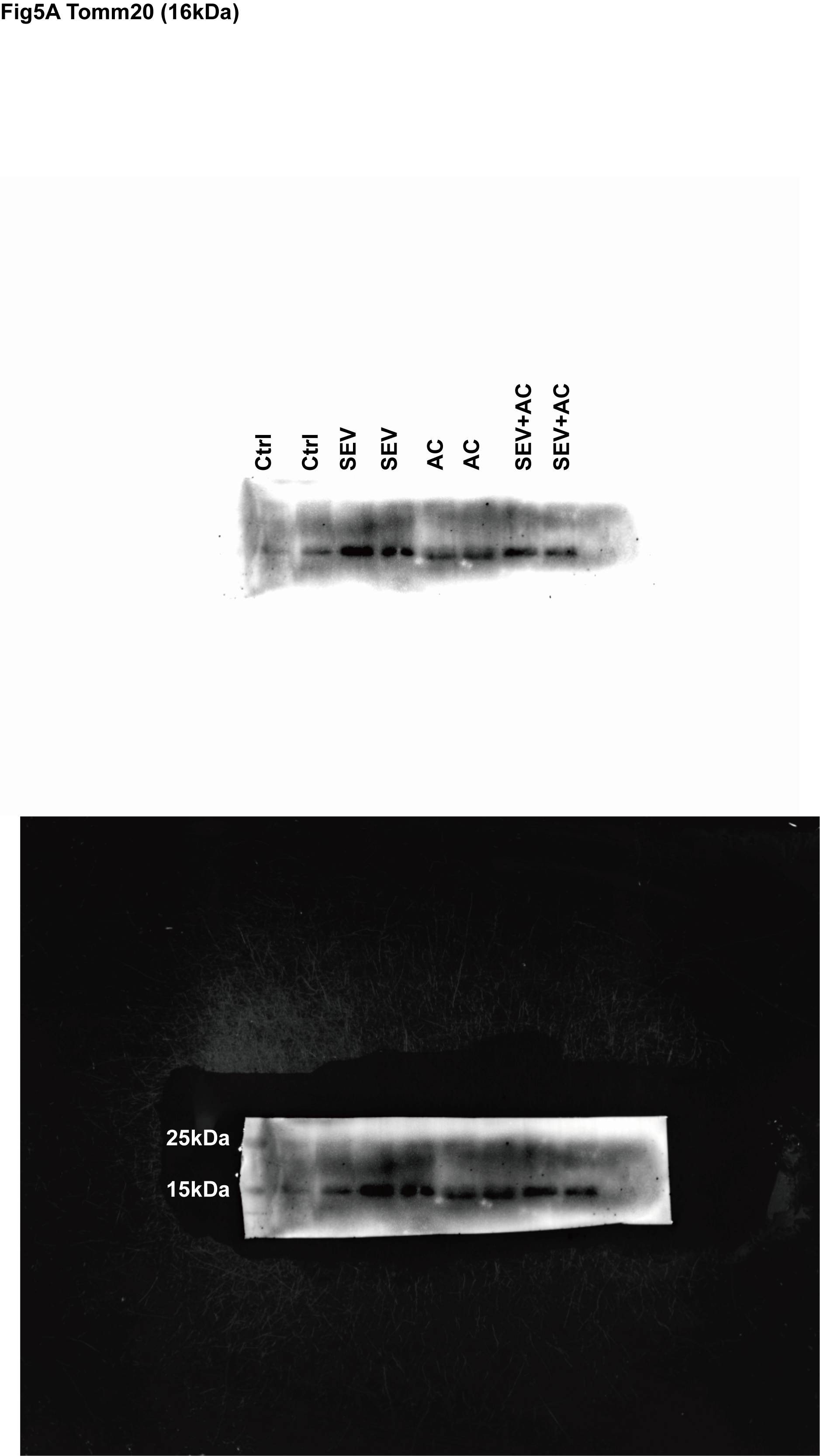

Supplement: S7 Raw images — (ZIP) [file pone.0280914.s012.zip › fig5A_raw_images/fig5A-Tomm20.tif]

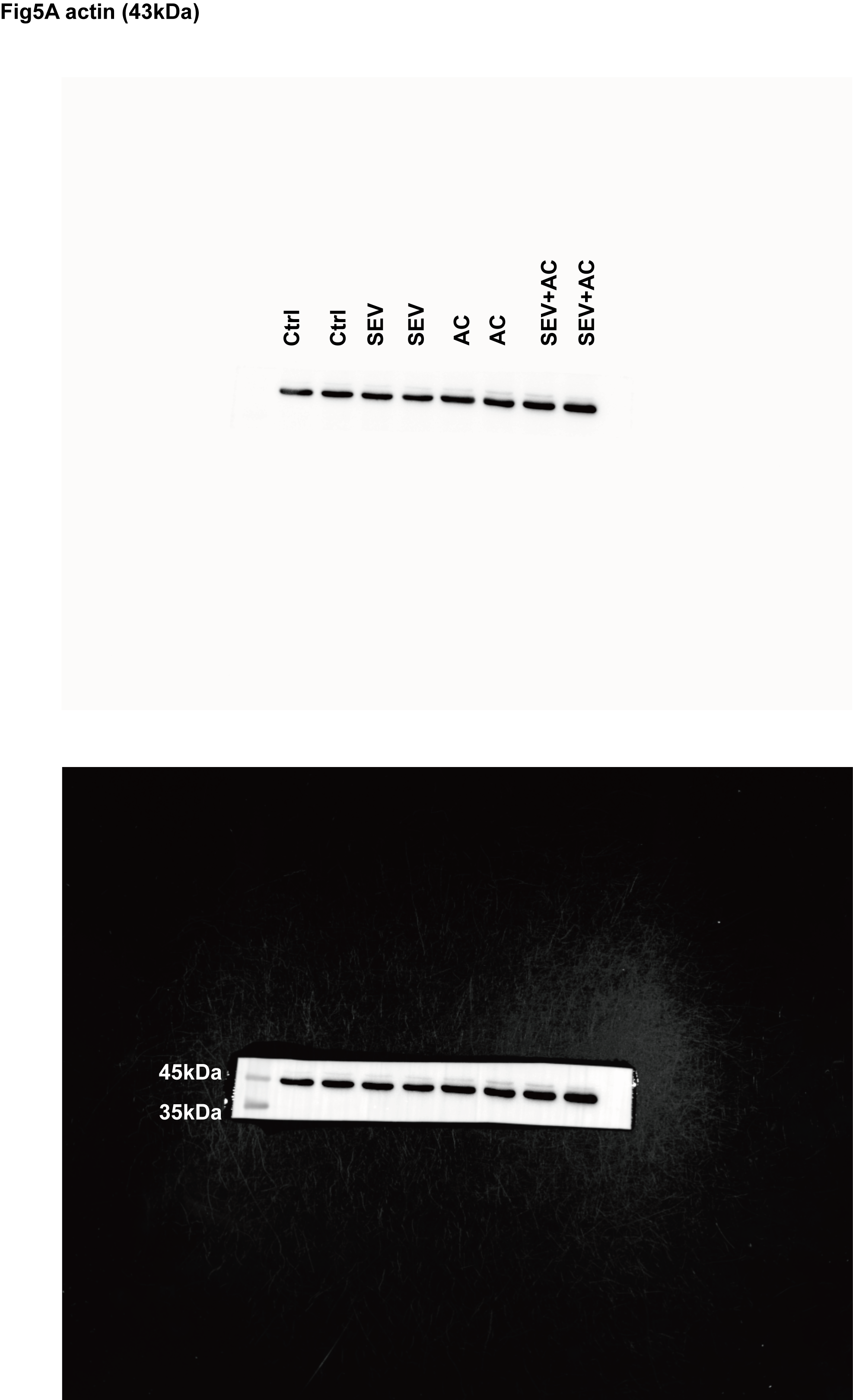

Supplement: S7 Raw images — (ZIP) [file pone.0280914.s012.zip › fig5A_raw_images/fig5A-actin.tif]

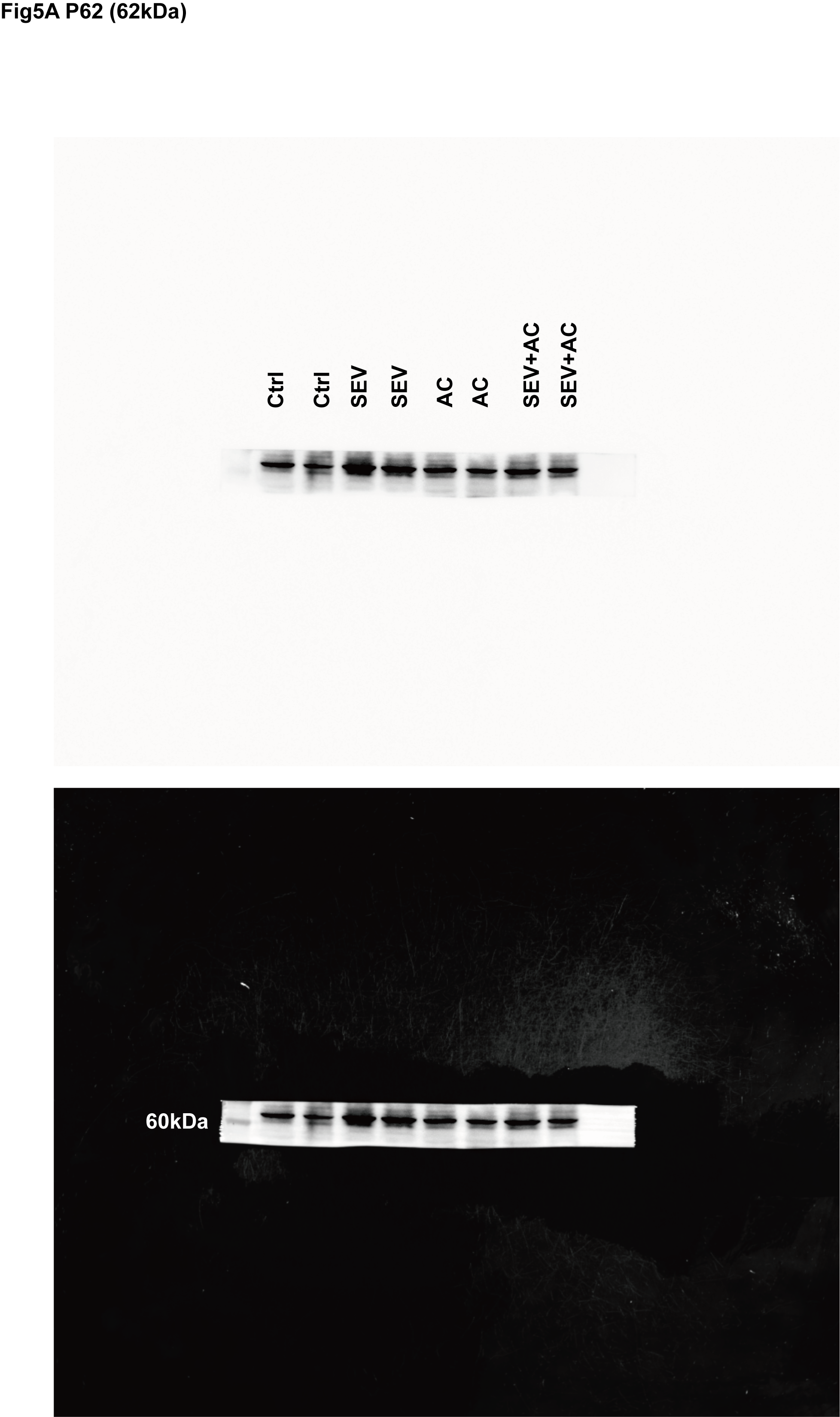

Supplement: S7 Raw images — (ZIP) [file pone.0280914.s012.zip › fig5A_raw_images/fig5A-p62.tif]
